# Modified frailty index versus risk analysis index for 30-day mortality after noncardiac surgery: a systematic review and meta-analysis

**DOI:** 10.3389/fmed.2026.1885620

**Published:** 2026-10-09

**Authors:** Xinrui Yin, Shijia Du

**Affiliations:** 1 Department of Anesthesiology, Aerospace Center Hospital, Beijing, China; 2 Department of VIP Dental Service, Peking University Stomatological Hospital, Beijing, China

**Keywords:** 30-day mortality, clinical frailty scale, discrimination, frailty, meta-analysis, modified frailty index, noncardiac surgery, risk analysis index

## Abstract

**Background:**

mFI and RAI are widely used in noncardiac surgical risk stratification, but they differ in content and data requirements. Because construct validity and outcome discrimination are distinct, we evaluated their prognostic discrimination for 30-day all-cause mortality without treating either instrument as a criterion-standard multidimensional frailty measure.

**Methods:**

This PRISMA-aligned, prospectively registered systematic review used two complementary evidence levels: tool-specific random-effects meta-analyses across independent cohorts and direct within-study comparison. Standalone AUCs were pooled on the logit scale using REML with Hartung–Knapp inference and 95% prediction intervals. Risk of bias was assessed with QUIPS. CFS was analyzed *post hoc* as secondary exploratory evidence.

**Results:**

Seventy-seven studies were included. For mFI, 39 independent cohorts yielded a pooled AUC of 0.634 (95% CI, 0.618–0.649; 95% prediction interval, 0.551–0.709). For RAI, 30 independent cohorts yielded 0.767 (95% CI, 0.737–0.795; 95% prediction interval, 0.579–0.888). Among 29 dual-tool studies, 21 had source-valid numeric comparisons in which RAI was higher, none had a source-valid numeric comparison in which mFI was higher, 2 supported RAI only qualitatively, and 6 could not be numerically classified; no paired meta-analysis was conducted because within-study covariance or delta-AUC standard errors were unavailable. Fourteen CFS studies were identified; 6 source-confirmed 30-day AUC cohorts yielded a *post hoc* exploratory AUC of 0.757 (95% CI, 0.618–0.857).

**Conclusions:**

RAI showed higher average prognostic discrimination than mFI, a pattern also observed in most directly comparative studies. Separate pooled AUCs are not a formal superiority test because case mix differs across studies. The analysis does not establish construct validity, and CFS evidence remains exploratory.

**Systematic Review Registration:**

https://www.crd.york.ac.uk/PROSPERO/view/CRD420261378778, identifier PROSPERO, CRD420261378778.

## 1 Introduction

Frailty is a state of heightened vulnerability to stressors, but it is not represented by a single universally accepted measurement model. Previous systematic reviews have associated frailty with adverse postoperative outcomes across surgical populations (1, 2). The physical phenotype operationalizes weakness, slowness, low activity, exhaustion, and weight loss, whereas the deficit-accumulation model summarizes the proportion of health deficits present (3–5). These approaches overlap clinically but are conceptually distinct, and neither should be conflated with multimorbidity alone (3, 6).

The modified Frailty Index (mFI) was developed within the deficit-accumulation tradition by mapping available surgical-registry variables to deficits and has been widely used in surgical outcomes research (4, 7, 8). Its compact versions, particularly mFI-5, are readily derived from routinely collected data. That economy has an important limitation: the retained content is heavily weighted toward comorbidity and functional dependence. Haren et al. argued that such reduced indices may measure multimorbidity or general vulnerability more directly than the multidomain frailty syndrome (9). This construct-validity concern is substantive. The present review does not treat mFI as a criterion-standard or comprehensive measure of frailty and does not attempt to revalidate it as such.

The Risk Analysis Index (RAI) was developed for surgical risk assessment and combines age, function, cognitive or living-context information, nutrition or weight loss, malignancy, dyspnea, and selected comorbidities, with different operationalizations across clinical and administrative versions (10, 11). The Clinical Frailty Scale (CFS) is a global clinical judgment scale based on overall fitness and frailty (12). Validation of any of these instruments for frailty-related classification or surgical risk stratification does not imply equivalent discrimination for a specific outcome. Construct validity and outcome discrimination are distinct measurement properties.

Thirty-day mortality provides a common, clinically consequential outcome for evaluating rank discrimination. Area under the receiver operating characteristic curve (AUC) quantifies whether a tool assigns higher risk to patients who experience the outcome than to those who do not; it does not establish construct validity, calibration, causal effects, or clinical net benefit (13–15).

We therefore used a two-level evidence framework. First, separate tool-specific meta-analyses characterized average discrimination and its distribution across the available independent surgical cohorts. Second, studies evaluating mFI and RAI in the same underlying population supplied the primary comparative context. Restricting eligibility to head-to-head studies would discard substantial evidence about absolute discrimination and transportability across surgical settings; conversely, separate pooled AUCs across different case mixes cannot serve as a formal indirect superiority test. We also retained CFS as secondary, *post hoc* exploratory evidence.

Our objective was to estimate the average standalone discrimination of mFI and RAI for 30-day all-cause mortality across independent noncardiac surgical cohorts and to interpret comparative performance primarily through dual-tool studies. Secondary objectives were to characterize heterogeneity and data quality, test sensitivity to variance provenance, overlap, risk of bias, and version dependence, and provide an explicit exploratory synthesis of CFS.

## 2 Methods

### 2.1 Protocol, reporting, and review scope

This systematic review and meta-analysis followed PRISMA 2020 guidance and the prospectively registered PROSPERO protocol (CRD420261378778) (16). Database-specific strategies, dates, and screening records are reported in Supplementary Appendix 1. Eligibility was not restricted to head-to-head studies; single-tool and dual-tool evidence were retained because they address complementary questions.

### 2.2 Eligibility criteria and two-level evidence framework

Eligible studies enrolled adults undergoing noncardiac surgery, assessed mFI (mFI-5 or mFI-11), RAI (original RAI, RAI-rev, or RAI-A), or CFS preoperatively or from baseline variables available before surgery, and reported 30-day all-cause mortality. Cohort and registry studies were eligible. Studies using only postoperative or intensive-care frailty assessment, cardiac surgery, severe burns, major polytrauma, noncomparable mortality windows, or only a frailty-containing multivariable model without a standalone tool estimate were not eligible for quantitative AUC synthesis.

Level 1 comprised separate tool-specific syntheses of standalone AUCs. Its purpose was to characterize the distribution and average discrimination of each instrument across independent cohorts, including evidence from studies evaluating only one tool. Level 2 comprised studies evaluating mFI and RAI within the same underlying study population. Single-tool evidence informed absolute discrimination and transportability; dual-tool evidence informed comparative interpretation.

The separately pooled tool-specific AUCs were intended to characterize overall discrimination across the available independent cohorts and were not interpreted as a formal indirect test of superiority. Comparative inference was therefore supported primarily by studies evaluating both instruments within the same study population.

### 2.3 Study selection and source verification

Two reviewers screened records and assessed full texts against prespecified criteria, resolving disagreements by consensus. Study-level data were reverified against the original source reports before the final quantitative synthesis. Quantitative inclusion required a source-confirmed standalone 30-day mortality AUC or c-statistic with a reported confidence interval or standard error, or exact sample size and mortality events sufficient for the specified variance approximation. The study selection process is summarized in Figure 1.

**Figure 1:**
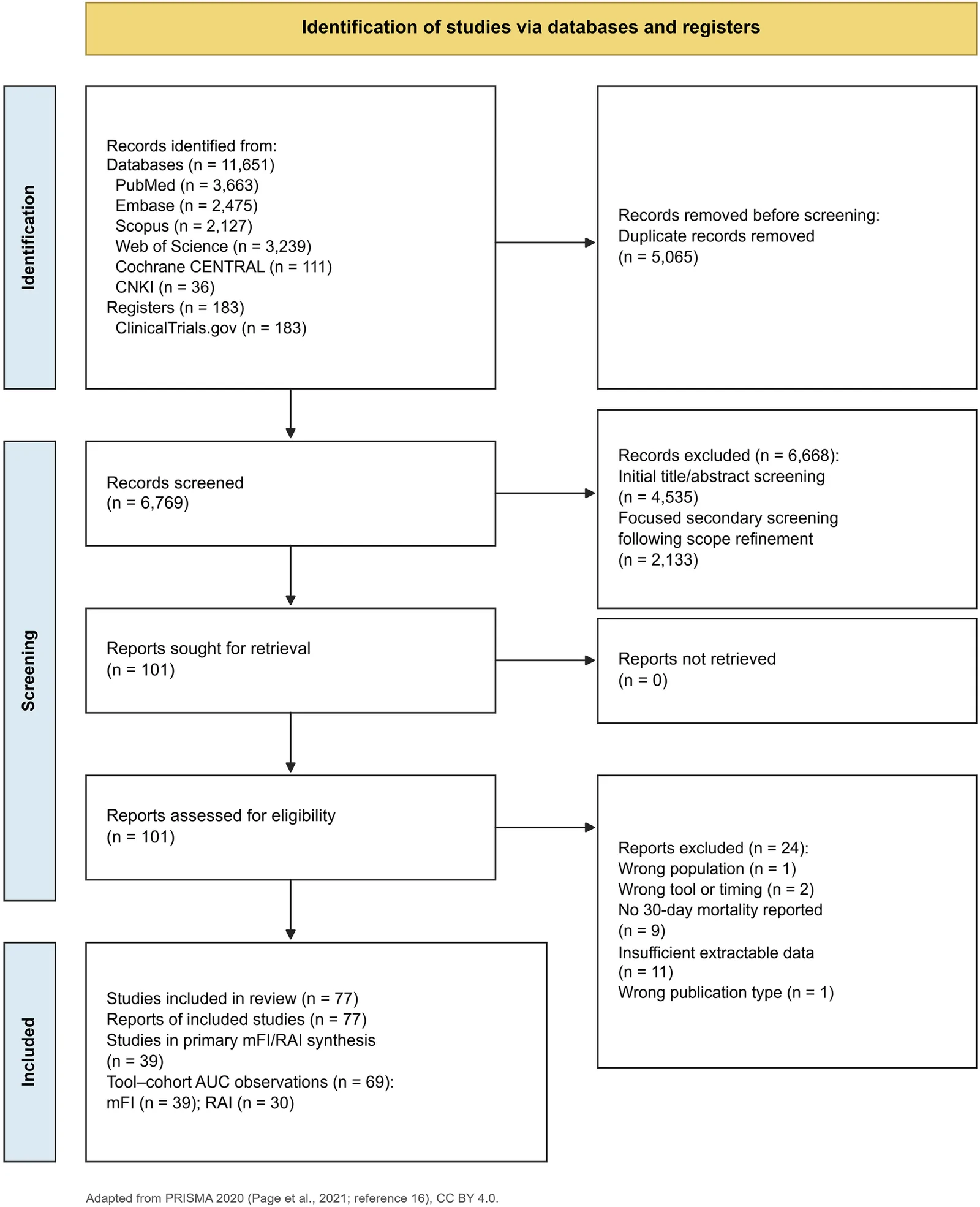
Study identification and selection. The PRISMA flow diagram and database-specific search accounting are provided in Supplementary Appendix 1. The final study universe contains 77 studies.

### 2.4 Data extraction, case mix, and cohort independence

The extraction captured study identity, surgical population and specialty, data source and years, sample size, mortality events when reported, instrument family and version, standalone AUC and uncertainty, direct-comparison information, variance provenance, overlap group, and QUIPS judgments. Surgical population, specialty, registry or health-system source, and study years were retained to characterize prognostic case mix and spectrum effects.

Each quantitative observation was mapped to an independent patient cohort. Nonoverlapping procedure cohorts within a publication could remain separate. When multiple versions of one tool family were evaluated in the same patients, the primary analysis used the source-designated or preferred version and did not average correlated AUCs. Alternative-version and cohort-clustered all-version analyses were used to assess sensitivity to this choice.

### 2.5 Outcome and effect measure

The outcome was 30-day all-cause mortality. The primary effect measure was the standalone tool-level AUC (equivalently, c-statistic). AUC was chosen because it was the most consistently available common measure of rank discrimination across heterogeneous populations. It was not used to infer calibration, threshold-specific performance, decision utility, construct validity, or a causal effect of frailty.

### 2.6 Risk of bias

Risk of bias was assessed with QUIPS across study participation, attrition, prognostic-factor measurement, outcome measurement, study confounding, and statistical analysis/reporting (17). Two reviewers adjudicated domain and overall ratings. For consistency of overall judgments, domain ratings were summarized using the following review-level rule: all domains Low, or only one Moderate outside D3/D4, was classified Low; 2–3 Moderate domains, or one Moderate in D3/D4, was classified Moderate; any High domain or at least four Moderate domains was classified High. Overall risk of bias was Low in 2 studies, Moderate in 52, and High in 23, with Hao 2025 rated High.

For standalone AUCs, case-mix or spectrum variation is conceptually different from confounding of an exposure–outcome association. AUC is a descriptive performance measure rather than a causal effect estimate, so conventional covariate adjustment is not directly analogous to adjustment of an odds ratio or hazard ratio. We nevertheless used the QUIPS Study Confounding domain to assess whether important prognostic structure was adequately addressed and interpreted AUCs in relation to study population and setting.

### 2.7 Statistical analysis

AUCs were transformed as logit(AUC) = ln[AUC/(1−AUC)] and back-transformed for presentation. For a reported 95% confidence interval wholly within 0 and 1, the logit-scale standard error was [logit(upper)−logit(lower)]/(2 × 1.96). A source-reported raw AUC standard error was converted by the delta method. For the two source-reported Wald intervals crossing 1, a raw-scale standard error was first calculated and then delta-converted. When only an AUC point estimate, exact total sample size, and exact mortality events were available, the Hanley–McNeil variance approximation was used; these records were explicitly labelled as having variance estimated from sample size and events (13).

Separate random-effects meta-analyses for mFI and RAI used restricted maximum likelihood on the logit-AUC scale. The pooled estimator was the inverse-variance random-effects mean. Hartung–Knapp inference used the unmodified q factor, with k−1 degrees of freedom (18, 19). The 95% confidence interval was *μ* ± t(k−1,0.975) × SEHK. The 95% prediction interval was μ ± t(k−1,0.975) × sqrt(*τ*^2^ + SEHK^2^), followed by inverse-logit transformation. I^2^ used the conventional fixed-effect Q and is reported with *τ*^2^ (20).

No formal difference between the separately pooled mFI and RAI estimates was tested. Direct studies were classified at study level as all eligible RAI versions numerically higher, all lower, mixed/equal, or not numerically classifiable. No paired meta-analysis was conducted because complete within-study covariance or a standard error for the AUC difference was unavailable.

### 2.8 Sensitivity and exploratory analyses

Sensitivity analyses included: an alternative eligible tool version for same-cohort version-sensitive clusters; a cohort-clustered CR1 sandwich analysis retaining all eligible versions; exclusion of every record whose variance was estimated from sample size and events; exclusion of High overall QUIPS studies; and an overlap-adjusted analysis retaining one representative per overlap group according to the documented selection rule. Subtype analyses were exploratory. These analyses characterize robustness but do not remove the underlying limitations.

CFS was retained as secondary evidence. Eligible CFS observations were pooled *post hoc* using the same conventional one-estimate-per-cohort method. This analysis was explicitly exploratory and was not used to rank CFS against mFI or RAI.

## 3 Results

### 3.1 Study universe and case mix

Seventy-seven studies met the systematic-review criteria (21–96): 29 evaluated both mFI and RAI, 18 evaluated mFI only, 16 evaluated RAI only, and 14 evaluated CFS only. The most common surgical categories were spine (17), general or abdominal surgery (13), orthopedic surgery excluding hip fracture (11), other (9), neurosurgery (7), hip fracture (6), vascular surgery (5), mixed surgery (4), urology (4), and colorectal surgery (1). Table 1 summarizes study classifications, populations, data sources, tool versions, cohort contributions, direct-comparison status, and QUIPS judgments.

**Table 1:** Characteristics of the included studies.

Characteristic	Category	No. of studies
**Frailty tool reported**		
	mFI + RAI in same cohort	29
	mFI alone	18
	RAI alone	16
	CFS	14
	Total	**77**
**Country or region**		
	United States	61
	United Kingdom	4
	China	2
	Australia	1
	Canada	1
	Norway	1
	Spain	1
	Turkey	1
	Yemen	1
	Principal study site not unambiguously specified	4
	Total	**77**
**Study design**		
	Retrospective registry-based cohort	57
	Prospective cohort	13
	Retrospective cohort, non-registry	5
	Observational cohort, timing not specified	2
	Total	**77**
**Data source or database**		
	ACS-NSQIP, sole source	48
	ACS-NSQIP + VASQIP combined cohort	3
	VASQIP alone	3
	Single-center prospective cohort	8
	Single-center retrospective cohort	7
	Multicenter non-registry cohort	4
	Other multicenter registry — ICES	2
	Integrated health system cohort/KPNC	1
	Other multicenter registry — NRD	1
	Total	**77**
**Surgical specialty**		
	Spine	17
	General or abdominal	13
	Orthopedic, excluding hip fracture	11
	Neurosurgery	7
	Hip fracture	6
	Vascular	5
	Urology	4
	Mixed surgical cohorts	4
	Other or unspecified	10
	Total	**77**
**Elective or emergency status**		
	Emergency	22
	Elective	13
	Mixed elective and emergency	7
	Not clearly reported	35
	Total	**77**
**Study sample size**		
	Median (IQR)	9,319 (1,255–61,441)
	Range	89–2,339,031
**Mean or median age, where available**		
	Studies with parseable numeric age	14
	Median (IQR), years	71.0 (68.0–80.8)
	Range, years	57.0–92.5

ACS-NSQIP, American College of Surgeons National Surgical Quality Improvement Program; CFS, Clinical Frailty Scale; ICES, Institute for Clinical Evaluative Sciences; IQR, interquartile range; KPNC, Kaiser Permanente Northern California; mFI, modified Frailty Index; NRD, Nationwide Readmissions Database; RAI, Risk Analysis Index; VASQIP, Veterans Affairs Surgical Quality Improvement Program.

Total participant count was not summed because of substantial overlap among registry-derived cohorts. Sample-size distribution is therefore reported as median, IQR, and range. Age was summarized only where a single parseable mean or median age was available.

Case mix varied materially by specialty, selected population, registry or health system, and calendar period. Among the 26 primary-analysis studies with completed case-mix source audit, comorbidity burden or cancer/procedure composition was reported in 26/26, sex in 25/26, functional dependence in 25/26, ASA or a comparable baseline-risk measure in 20/26, and age in 19/26. For the QUIPS Study Confounding domain, 51 studies were Low and 26 were Moderate; none was High. Across all QUIPS domains, overall risk of bias was Low in 2 studies, Moderate in 52, and High in 23. This distribution, together with the heterogeneous population descriptions in Table 1, supports interpretation of pooled AUCs as averages across a broad prognostic spectrum rather than intrinsic constants of the instruments.

### 3.2 Level 1: tool-specific pooled discrimination

For mFI, 39 independent cohorts contributed to the primary analysis. The pooled AUC was 0.634 (95% CI, 0.618–0.649), with *τ*^2^ = 0.027706, I^2^ = 98.8%, and a 95% prediction interval of 0.551–0.709. For RAI, 30 independent cohorts contributed. The pooled AUC was 0.767 (95% CI, 0.737–0.795), with *τ*^2^ = 0.176481, I^2^ = 99.8%, and a 95% prediction interval of 0.579–0.888 (Table 2; Figure 2). These separate pools describe average discrimination within each available evidence base and are not a formal indirect superiority test.

**Table 2:** Primary and sensitivity analyses of pooled AUC estimates for mFI and RAI.

**Analysis**	**Tool**	**Cohorts** **(k)**	**AUC** **estimates**	**Pooled** **AUC**	**95% CI**	**τ²**	**I² (%)**	**95% PI**
Primary analysis	mFI	39	39	0.634	0.618–0.649	0.027706	98.8	0.551–0.709
Primary analysis	RAI	30	30	0.767	0.737–0.795	0.176481	99.8	0.579–0.888
Excluding estimated variances	mFI	32	32	0.632	0.615–0.649	0.028677	99.0	0.547–0.710
Excluding estimated variances	RAI	29	29	0.769	0.738–0.797	0.181090	99.8	0.578–0.890
Overlap-adjusted analysis	mFI	28	28	0.628	0.607–0.648	0.033266	89.6	0.535–0.713
Overlap-adjusted analysis	RAI	23	23	0.751	0.714–0.786	0.186474	99.7	0.547–0.883
Low/Moderate QUIPS only	mFI	37	37	0.633	0.617–0.648	0.027159	98.8	0.551–0.708
Low/Moderate QUIPS only	RAI	29	29	0.767	0.736–0.795	0.178086	99.8	0.577–0.888
Alternative-version analysis	mFI	39	39	0.634	0.618–0.649	0.027706	98.8	0.551–0.709
Alternative-version analysis	RAI	30	30	0.763	0.733–0.790	0.166791	99.8	0.579–0.883
Clustered all-version analysis	mFI	39	39	0.634	0.619–0.649	0.027706	98.8	0.551–0.709
Clustered all-version analysis	RAI	30	35	0.763	0.730–0.793	0.175282	99.8	0.574–0.885

AUC, area under the receiver operating characteristic curve; CI, confidence interval; PI, prediction interval; mFI, modified Frailty Index; RAI, Risk Analysis Index; QUIPS, Quality in Prognosis Studies; REML, restricted maximum likelihood.

Cohorts (k) denotes independent cohorts within each tool-specific analysis; AUC estimates denotes the number of tool-version observations. Primary analyses use one preferred version per cohort. Separate tool-specific pools are not a formal between-tool superiority test.

Estimates were pooled on the logit-AUC scale using REML with Hartung–Knapp inference and back-transformed. τ² is on the logit-AUC scale. The clustered all-version analysis instead uses cohort-clustered CR1 sandwich inference; within-cohort covariances are unknown.

Estimated-variance exclusion removes seven mFI and one RAI observations whose variances were approximated from sample size and mortality events. Overlap adjustment retains one representative per overlap group (Supplementary Appendix 3). Low/Moderate QUIPS analyses exclude High-risk studies. Alternative-version analysis substitutes eligible alternative versions in version-sensitive cohorts.

**Figure 2:**
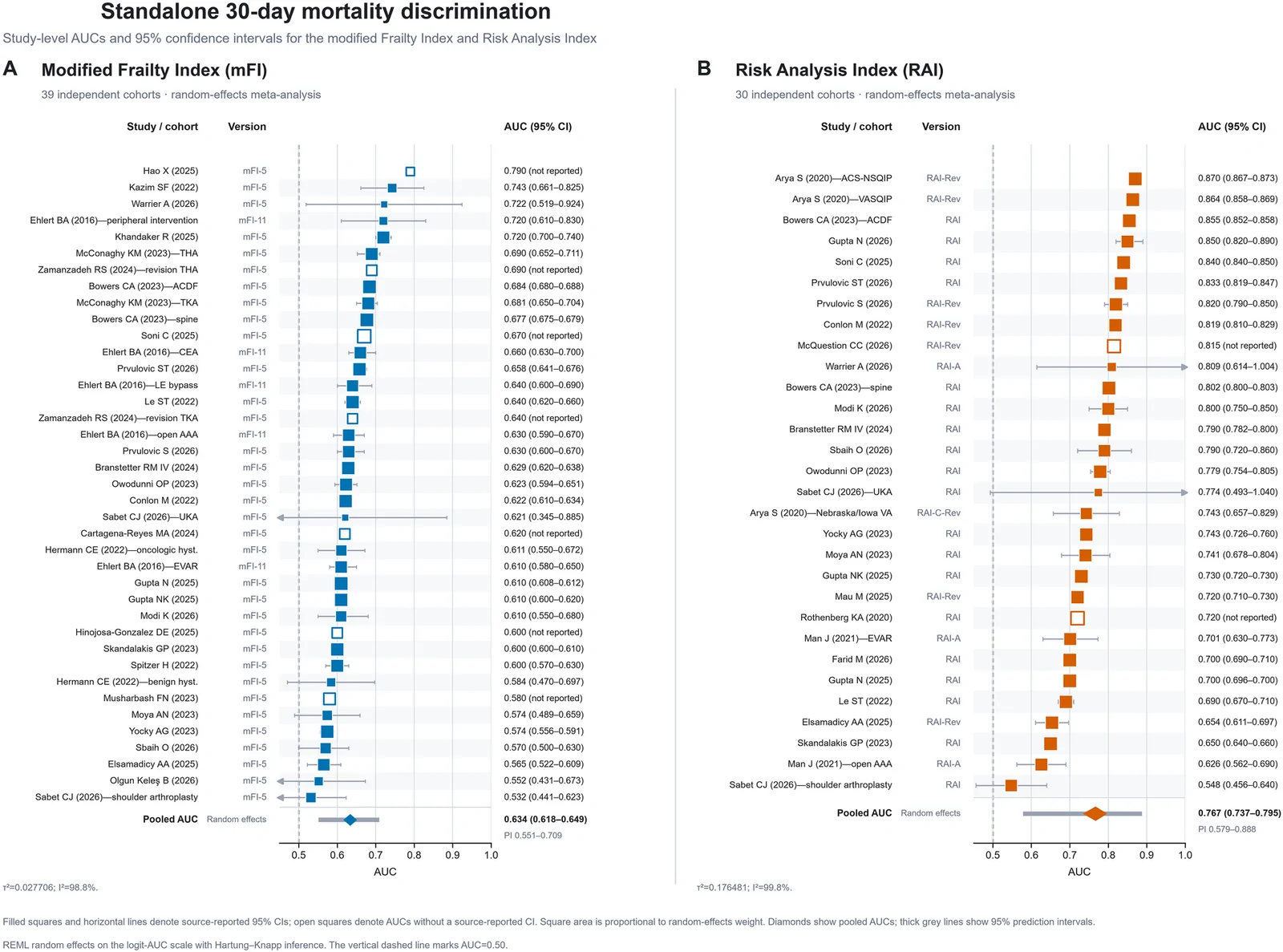
Independent–cohort forest plots of standalone AUC for 30-day all–cause mortality. Panels (**A and B**) display the 39 mFI and 30 RAI primary observations, respectively. Squares are study estimates; horizontal lines are source confidence intervals. The diamond is the pooled AUC and its 95% CI; the thick gray line is the 95% prediction interval. No between-tool test is implied.

### 3.3 Level 2: direct mFI-versus-RAI evidence

Twenty-nine dual-tool studies informed direct comparative interpretation. At the study level, 21 had source-valid numeric comparisons in which RAI was higher, none had a source-valid numeric comparison in which mFI was higher, 2 supported RAI only qualitatively, and 6 were not numerically classifiable for the comparable standalone 30-day all-cause mortality outcome. Figure 3 presents the direct evidence; no paired meta-analysis was performed because within-study covariance or standard errors for AUC differences were unavailable.

**Figure 3:**
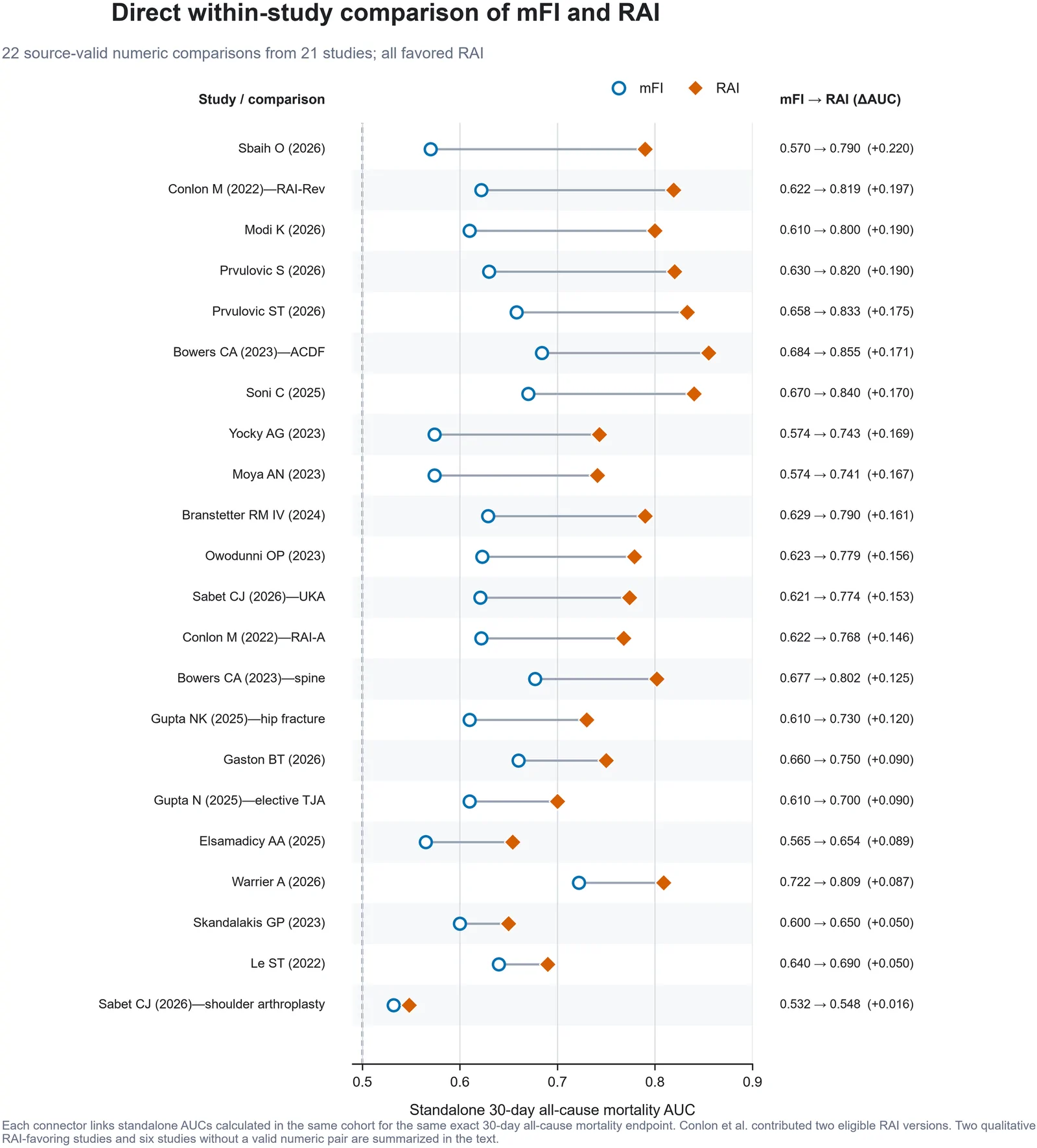
Direct mFI-versus-RAI evidence. The plot shows 22 source-valid numeric comparisons from 21 studies; Conlon et al. contributed two eligible RAI versions. Open blue circles represent mFI and orange diamonds represent RAI. Horizontal connectors link standalone 30-day all-cause mortality AUCs within the same cohort; ΔAUC = AUC(RAI)−AUC(mFI). No paired confidence intervals or pooled paired effect are displayed. Across all 29 dual-tool studies, 21 had numerically higher RAI AUCs, none had numerically higher mFI AUCs, 2 supported RAI only qualitatively, and 6 were not numerically classifiable. The latter eight studies are described in the text and are not plotted.

Twenty-two source-valid numeric comparison rows represented 21 studies because Conlon et al. reported two eligible RAI versions. Sabet CJ et al. (2026) contributed the corrected 30-day mortality pair (*N* = 11,965; 19 deaths): mFI-5 AUC 0.532 (95% CI, 0.441–0.623) and RAI AUC 0.548 (95% CI, 0.456–0.640), for a within-study difference of +0.016. Mau 2025 and Farid 2026 supported RAI only qualitatively because an exact standalone mFI mortality AUC was not printed. Courville 2024 was not numerically classifiable and was excluded from both quantitative pools because its source endpoint was in-hospital death at the index admission or a readmission within 30 days, while the results table used the composite label mortality or comfort care; this did not meet the quantitative-synthesis criterion requiring a source-confirmed standalone AUC for 30-day all-cause mortality.

### 3.4 CFS evidence

Fourteen CFS studies were present in the review universe. After exact mortality-window verification, six independent cohorts had source-confirmed standalone 30-day mortality AUCs with usable uncertainty. Narula 2020 was excluded because its AUC of 0.699 (95% CI, 0.651–0.747) was explicitly for one-year mortality and no standalone 30-day AUC was reported. Zaib 2023 was excluded because its AUC of 0.710 (95% CI, 0.650–0.760) was reported for unspecified mortality in a study presenting both 30-day and 2-year outcomes; the ROC itself was not time-labeled and therefore did not satisfy the 30-day source-confirmation criterion used for this quantitative synthesis. In the *post hoc* exploratory meta-analysis, the pooled CFS AUC was 0.757 (95% CI, 0.618–0.857), with *τ*^2^ = 0.254567, I^2^ = 77.4%, and a 95% prediction interval of 0.421–0.930. No eligible direct same-cohort CFS-versus-mFI or CFS-versus-RAI comparison was identified for the comparable 30-day discrimination outcome; accordingly, CFS was not formally ranked against either tool.

### 3.5 Sensitivity and subtype analyses

Results remained directionally consistent across the data-quality and dependence analyses, while interval width and heterogeneity continued to show substantial uncertainty (Table 2). Excluding estimated variances yielded pooled AUCs of 0.632 (95% CI, 0.615–0.649; prediction interval, 0.547–0.710) for 32 mFI cohorts and 0.769 (95% CI, 0.738–0.797; prediction interval, 0.578–0.890) for 29 RAI cohorts. Seven mFI and one RAI records were excluded by that rule; source-reported confidence intervals or standard errors were retained when available.

The overlap-adjusted results were 0.628 (95% CI, 0.607–0.648; prediction interval, 0.535–0.713) for 28 mFI cohorts and 0.751 (95% CI, 0.714–0.786; prediction interval, 0.547–0.883) for 23 RAI cohorts. Restriction to Low or Moderate overall QUIPS studies yielded 0.633 (95% CI, 0.617–0.648; prediction interval, 0.551–0.708) for 37 mFI cohorts and 0.767 (95% CI, 0.736–0.795; prediction interval, 0.577–0.888) for 29 RAI cohorts.

Alternative-version analysis did not change the mFI estimate and yielded an RAI AUC of 0.763 (95% CI, 0.733–0.790; prediction interval, 0.579–0.883). The cohort-clustered all-version analysis yielded 0.634 (95% CI, 0.619–0.649; prediction interval, 0.551–0.709) for mFI and 0.763 (95% CI, 0.730–0.793; prediction interval, 0.574–0.885) for RAI. In a one-study sensitivity analysis addressing the confidence-interval source conflict in Sbaih et al., the abstract-reported intervals [mFI-5 0.570 [95% CI, 0.530–0.610]; RAI 0.790 [95% CI, 0.750–0.830]] were substituted for the primary endpoint-linked main-results intervals [mFI-5 0.570 [95% CI, 0.500–0.630]; RAI 0.790 [95% CI, 0.720–0.860]]. This substitution changed the mFI pooled AUC from 0.634 to 0.633 (95% CI, 0.618–0.649; *τ*^2^ = 0.028431; I^2^ = 98.8%; prediction interval, 0.550–0.710) and left the rounded RAI pooled AUC at 0.767 (95% CI, 0.737–0.795; *τ*^2^ = 0.175691; I^2^ = 99.8%; prediction interval, 0.579–0.887). This did not alter the interpretation. Complete subtype estimates are provided in Supplementary Appendix 4 and are exploratory, particularly where k was small.

## 4 Discussion

### 4.1 Principal findings and two-level interpretation

This synthesis found higher average 30-day mortality discrimination for RAI than for mFI across the available independent surgical cohorts. That pattern was also observed in the direct evidence: 21 of 29 dual-tool studies had source-valid numeric comparisons in which RAI was higher, none had a source-valid numeric comparison in which mFI was higher, 2 supported RAI only qualitatively, and 6 were not numerically classifiable. The direct same-study evidence is the comparative anchor because both instruments are evaluated in the same underlying population. The separate tool-specific pools instead describe average performance and transportability across each tool's evidence base.

This distinction is essential. Surgical specialty, baseline risk, emergency or elective composition, instrument version, registry coding, missingness, and analytic specification differ across studies. Consequently, the difference between the pooled AUCs is not a formal indirect superiority test. Same-study comparisons reduce major between-study case-mix differences, but they do not eliminate differences caused by missing data, version selection, or analysis specification, and the absence of paired covariance precluded a formal paired meta-analysis.

### 4.2 Construct validity is distinct from prognostic discrimination

An important conceptual consideration is that the mFI label should not be interpreted as evidence that the instrument fully captures the multidimensional frailty construct. mFI arose from deficit accumulation and has pragmatic value in registry research (4, 7, 8), but its reduced content is comorbidity-heavy. Haren et al. argued that many such surgical indices more closely represent multimorbidity than multidimensional frailty (9). Vetrano et al. likewise showed that frailty and multimorbidity commonly coexist but are distinct (6). The Fried phenotype and a standard deficit-accumulation index capture different conceptual traditions (3–5).

Our analysis neither adjudicates whether mFI is a definitive frailty measure nor revalidates RAI or CFS as frailty constructs. We do not use predictive performance to validate the frailty construct. The present analysis evaluates prognostic discrimination for one common outcome. The observed difference in prognostic discrimination is compatible with the possibility that broader vulnerability content contributes additional prognostic information, but the present meta-analysis was not designed to establish construct validity or identify the mechanism underlying the difference.

### 4.3 Why single-tool evidence and AUC remain informative

A head-to-head-only review would answer the direct comparative question but would discard studies that inform absolute discrimination and transportability across specialties and data environments. Our two-level framework preserves both uses without conflating them. Single-tool cohorts characterize how widely performance varies in practice; dual-tool studies provide the more internally comparable evidence for relative performance.

Similarly, prior validation does not imply that two tools have equivalent discrimination for 30-day mortality. AUC measures rank discrimination for this outcome. It does not assess calibration, thresholds, net benefit, workflow burden, or construct validity (13–15). Tool selection therefore cannot be made from AUC alone, but comparing source-valid standalone AUCs remains clinically meaningful when the estimand and limits are explicit.

### 4.4 Heterogeneity, case mix, and mitigation

Heterogeneity was very high for both primary pools and cannot be considered resolved. The broad prediction intervals show that discrimination expected in a future population may differ substantially from the average. Likely contributors include specialty, baseline mortality risk, emergency versus elective composition, tool version, registry definitions, coding, and health-system context. AUC also varies with the case-mix spectrum: a more heterogeneous population can show different discrimination even when the underlying model is unchanged.

We characterized rather than eliminated these limitations through random-effects modeling, prediction intervals, independent-cohort handling, a variance-provenance sensitivity analysis, assessment of potential database overlap, Low/Moderate QUIPS restriction, subtype analyses, and direct same-study comparison. These measures reduce avoidable analytic distortion and display uncertainty; they do not make the underlying populations homogeneous.

### 4.5 CFS

CFS is a global, clinician-judgment measure designed to summarize overall fitness and frailty; unlike registry-derived indices, it can be applied at the bedside without reconstructing a deficit score and may therefore be practical for routine clinical screening (12). The review identified 14 CFS studies, of which 6 provided source-confirmed standalone 30-day mortality AUCs for the *post hoc* exploratory synthesis. The two other previously considered AUC-ready reports were excluded because Narula 2020 reported a one-year AUC and Zaib 2023 did not identify the mortality time window for its ROC AUC. The pooled estimate suggests potentially useful discrimination, but the available data do not permit a valid ranking of CFS relative to either instrument, because no eligible same-cohort comparison used the comparable 30-day standalone AUC outcome. Its practical bedside characteristics may make it attractive for routine screening, but ease of use and implementation burden were not outcomes evaluated in this review. Future direct studies should compare CFS, mFI, and RAI within the same cohorts and report discrimination, calibration, threshold-specific metrics, missingness, workflow feasibility, and decision utility.

### 4.6 Strengths and limitations

Strengths include source verification of study-level data, use of independent patient cohorts rather than publication or tool-version rows, explicit handling of multiple versions applied to the same cohort, assessment of potential registry overlap, and QUIPS assessment of all 77 studies. The direct-comparison evidence was assembled at the study level rather than inferred from the quantitative meta-analysis subset.

Several limitations remain. First, between-study heterogeneity and broad prediction intervals limit the portability of average AUCs; random effects, prediction intervals, subtype analyses, and direct comparisons characterize but do not remove this limitation. Second, registry overlap cannot be excluded perfectly; a source-based overlap assessment and sensitivity analysis reduced avoidable duplication. Third, uncertainty was estimated from sample size and events for some records; provenance was recorded and all such records were excluded in sensitivity analysis. Fourth, 23 studies were at High overall risk of bias; QUIPS and the Low/Moderate restriction show the effect of excluding them but do not repair primary-study limitations.

Fifth, multiple tool versions, variable coding, and missingness create dependence and measurement heterogeneity; we used one preferred version per independent cohort and performed alternative-version and cohort-clustered analyses. Sixth, case-mix differences prevent the separate pools from serving as a causal or formal indirect comparison; comparative interpretation therefore relies primarily on same-study evidence. Seventh, AUC does not describe calibration or clinical utility (14, 15). Eighth, the mFI label does not settle construct validity, and this review cannot determine why discrimination differs. Finally, CFS synthesis was *post hoc* and remains exploratory.

## 5 Conclusions

RAI showed higher average prognostic discrimination for 30-day mortality than mFI across the available independent cohorts, and most direct comparative studies showed the same numerical direction. Heterogeneous case mix prevents the separate pooled AUCs from being interpreted as a formal superiority test, and the review does not establish that either tool is a criterion-standard measure of multidimensional frailty. mFI remains useful for registry-derived population research, while RAI may provide more informative individual risk stratification when its data requirements and workflow are feasible. CFS evidence is promising but *post hoc* and exploratory, and is insufficient for formal comparative ranking. Future same-cohort studies should evaluate discrimination together with calibration, thresholds, missingness, implementation burden, and decision utility.

## Data Availability

The original contributions presented in the study are included in the article/Supplementary Material, further inquiries can be directed to the corresponding author.

## References

[1] Lin HS Watts JN Peel NM Hubbard RE. Frailty and post-operative outcomes in older surgical patients: a systematic review. BMC Geriatr. (2016) 16(1):157. 10.1186/s12877-016-0329-8 27580947 PMC5007853

[2] Oakland K Nadler R Cresswell L Jackson D Coughlin P. Systematic review and meta-analysis of the association between frailty and outcome in surgical patients. Ann R Coll Surg Engl. (2016) 98(2):80–5. 10.1308/rcsann.2016.0048 26741674 PMC5210486

[3] Fried LP Tangen CM Walston J Newman AB Hirsch C Gottdiener J. Frailty in older adults: evidence for a phenotype. J Gerontol A Biol Sci Med Sci. (2001) 56(3):M146–56. 10.1093/gerona/56.3.M146 11253156

[4] Rockwood K Mitnitski A. Frailty in relation to the accumulation of deficits. J Gerontol A Biol Sci Med Sci. (2007) 62(7):722–7. 10.1093/gerona/62.7.722 17634318

[5] Searle SD Mitnitski A Gahbauer EA Gill TM Rockwood K. A standard procedure for creating a frailty index. BMC Geriatr. (2008) 8(1):24. 10.1186/1471-2318-8-24 18826625 PMC2573877

[6] Vetrano DL Palmer K Marengoni A Marzetti E Lattanzio F Roller-Wirnsberger R. Frailty and multimorbidity: a systematic review and meta-analysis. J Gerontol A Biol Sci Med Sci. (2019) 74(5):659–66. 10.1093/gerona/gly110 29726918

[7] Velanovich V Antoine H Swartz A Peters D Rubinfeld I. Accumulating deficits model of frailty and postoperative mortality and morbidity: its application to a national database. J Surg Res. (2013) 183(1):104–10. 10.1016/j.jss.2013.01.021 23415494

[8] Subramaniam S Aalberg JJ Soriano RP Divino CM. New 5-factor modified frailty index using American college of surgeons NSQIP data. J Am Coll Surg. (2018) 226(2):173–181e8. 10.1016/j.jamcollsurg.2017.11.005 29155268

[9] Haren A Lal R Walker D Nair R Partridge J Dhesi J. Frailty assessment in older urological patients prior to surgery: a systematic review and narrative synthesis. Ther Adv Urol. (2020) 12:1756287220916614. 10.1177/1756287220916614 32435276 PMC7225795

[10] Hall DE Arya S Schmid KK Blaser C Carlson MA Bailey TL. Development and initial validation of the risk analysis index for measuring frailty in surgical populations. JAMA Surg. (2017) 152(2):175–82. 10.1001/jamasurg.2016.4202 27893030 PMC7140150

[11] Arya S Varley P Youk A Borrebach JD Perez S Massarweh NN. Recalibration and external validation of the risk analysis index. Ann Surg. (2020) 272(6):996–1005. 10.1097/sla.0000000000003276 30907757 PMC8785437

[12] Rockwood K Song X MacKnight C Bergman H Hogan DB McDowell I. A global clinical measure of fitness and frailty in elderly people. Can Med Assoc J. (2005) 173(5):489–95. 10.1503/cmaj.050051 PMC1188185 16129869

[13] Hanley JA McNeil BJ. The meaning and use of the area under a receiver operating characteristic (ROC) curve. Radiology. (1982) 143(1):29–36. 10.1148/radiology.143.1.7063747 7063747

[14] Collins GS Reitsma JB Altman DG Moons KGM. Transparent reporting of a multivariable prediction model for individual prognosis or diagnosis (TRIPOD): the TRIPOD statement. Ann Intern Med. (2015) 162(1):55–63. 10.7326/m14-0697 25560714

[15] Vickers AJ Elkin EB. Decision curve analysis: a novel method for evaluating prediction models. Med Decis Making. (2006) 26(6):565–74. 10.1177/0272989x06295361 17099194 PMC2577036

[16] Page MJ McKenzie JE Bossuyt PM Boutron I Hoffmann TC Mulrow CD. The PRISMA 2020 statement: an updated guideline for reporting systematic reviews. Br Med J. (2021) 372:n71. 10.1136/bmj.n71 PMC8005924 33782057

[17] Hayden JA Van Der Windt DA Cartwright JL Côté P Bombardier C. Assessing bias in studies of prognostic factors. Ann Intern Med. (2013) 158(4):280–6. 10.7326/0003-4819-158-4-201302190-00009 23420236

[18] Hartung J Knapp G. A refined method for the meta-analysis of controlled clinical trials with binary outcome. Stat Med. (2001) 20(24):3875–89. 10.1002/sim.1009 11782040

[19] Balduzzi S Rücker G Schwarzer G. How to perform a meta-analysis with R: a practical tutorial. Evidence Based Mental Health. (2019) 22(4):153–60. 10.1136/ebmental-2019-300117 31563865 PMC10231495

[20] Higgins JPT Thompson SG Deeks JJ Altman DG. Measuring inconsistency in meta-analyses. Br Med J. (2003) 327(7414):557–60. 10.1136/bmj.327.7414.557 PMC192859 12958120

[21] Talukder R Taghlabi KM Khan R Melhem M McManus R Hassan T. Predictive factors for postoperative complications in nerve grafting neurorrhaphies: a multispecialty analysis using NSQIP data. Clin Neurol Neurosurg. (2025) 254:108918. 10.1016/j.clineuro.2025.108918 40318461

[22] Ghanekar SD Serrato P Brown EDL Sadeghzadeh S Maity A Khalid SI. Assessing combined effects of RAI, GNRI, and anemia on morbidity and mortality in elderly patients after subdural hematoma evacuation. Clin Neurol Neurosurg. (2026) 263:109340. 10.1016/j.clineuro.2026.109340 41655319

[23] Khandaker R Wongtangman K Frank M Borngaesser F Smith RV Nie L. Development of the anesthesia risk assessment score (ARAS) for postoperative mortality and adverse discharge to a nursing facility. J Clin Anesth. (2025) 106:111918. 10.1016/j.jclinane.2025.111918 40592217

[24] Gupta N Sasaki J Koltenyuk V Park A Chmait HR Perugini A. The risk analysis index as a predictor of 30-day mortality for elderly obese patients undergoing elective total joint arthroplasty. J Orthop. (2025) 65:178–84. 10.1016/j.jor.2025.05.009 40487330 PMC12142224

[25] Levy I Finkelstein M Bilal KH Palese M. Modified frailty index associated with clavien-dindo IV complications in robot-assisted radical prostatectomies: a retrospective study. Urol Oncol: Semin Orig Invest. (2017) 35(6):425–31. 10.1016/j.urolonc.2017.01.005 28190748

[26] Sabet CJ Jain B Hammadeh BM Liu J. Predicting postoperative risk in adult men undergoing ankle fracture surgery: development of a novel composite frailty score. Surgeon. (2026) 24:320–7 S1479-666X(26)00018-1. 10.1016/j.surge.2026.02.009 41775619

[27] Thommen R Kazim SF Cole KL Olson GT Shama L Lovato CM. Worse pituitary adenoma surgical outcomes predicted by increasing frailty, not age. World Neurosurg. (2022) 161:e347–54. 10.1016/j.wneu.2022.02.002 35134588

[28] Branstetter RM Owodunni OP Courville EN Courville JT Gagliardi TA Conti JT. The weight of frailty in neurosurgery patients: analyzing the combined effect of frailty and body mass index on 30-day postoperative mortality. World Neurosurg. (2024) 184:e449–59. 10.1016/j.wneu.2024.01.145 38310945

[29] Prvulovic ST Zoghi S Gajjar A Sabet CJ Covell MM Pahwa B. Predictive value of geriatric nutritional risk index and risk analysis index for post-operative outcomes in spine surgery patients: a comprehensive analysis. Global Spine J. (2026) 16(2):1177–91. 10.1177/21925682251375072 40876846 PMC12394237

[30] Sbaih O Gupta NK Ekowa D Prvulovic S Modi K Miller M. Comparison of the risk analysis index and the modified 5-item frailty index in predicting 30-day morbidity and mortality after occipitocervical fusion. Global Spine J. (2026) 16(4):1917–26. 10.1177/21925682251392178 41175085 PMC12579592

[31] Wu HHL Van Mierlo R McLauchlan G Challen K Mitra S Dhaygude AP. Prognostic performance of clinical assessment tools following hip fracture in patients with chronic kidney disease. Int Urol Nephrol. (2021) 53(11):2359–67. 10.1007/s11255-021-02798-7 33686533 PMC7939449

[32] Chen X Dang Y Zhang Q Ma Y Yao L Wang H. MFI-11 in Chinese elderly esophageal cancer patients with postoperative adverse outcomes. BMC Geriatr. (2024) 24(1):677. 10.1186/s12877-024-05281-x 39138432 PMC11323644

[33] Gilan WM Obadiel YA Hashem AF Aiash SA Jowah HM. Frailty as a predictor of postoperative morbidity and mortality in older patients undergoing emergency laparotomy: a prospective cohort study in Yemen. BMC Geriatr. (2025) 25(1):567. 10.1186/s12877-025-06222-y 40745302 PMC12312323

[34] Hao X Xu Z Guo Y Liu J Tian J Cao F. Brief preoperative frailty predicts postoperative adverse outcomes in older patients with radical esophageal cancer surgery. BMC Geriatr. (2025) 25(1):781. 10.1186/s12877-025-06452-0 41094653 PMC12523159

[35] Olgun Keleş B Şahin Y Tekir Yilmaz E Altinbaş A. Predictors of postoperative survival and mortality in nonagenarian surgical patients: a retrospective cohort study. BMC Surg. (2026) 26(1):232. 10.1186/s12893-026-03604-w PMC13037167 41731474

[36] Sabet CJ Jain B Hammadeh BM Kikhia A Alfawareh MD. Risk analysis index demonstrates superior predictive performance compared to traditional frailty metrics in elderly female patients undergoing inpatient total shoulder arthroplasty. Arthroplasty. (2026) 8:18. 10.1186/s42836-025-00366-3 41782056 PMC12961877

[37] Modi K Sbaih O Sasaki J Koltenyuk V Gupta N Patel H. Risk analysis index as a predictor of mortality and failure to rescue in patients undergoing distal femur fracture fixation. Indian J Orthop. (2026) 60:987–94. 10.1007/s43465-025-01610-3 42027345 PMC13100094

[38] Ikram A Norrish AR Marson BA Craxford S Gladman JRF Ollivere BJ. Can the clinical frailty scale on admission predict 30-day survival, postoperative complications, and institutionalization in patients with fragility hip fracture? A cohort study of 1,255 patients. Bone Joint J. (2022) 104-B(8):980–6. 10.1302/0301-620X.104B8.BJJ-2020-1835.R2 35909371 PMC9948448

[39] Narula S Lawless A D’Alessandro P Jones CW Yates P Seymour H. Clinical frailty scale is a good predictor of mortality after proximal femur fracture. Bone Joint Open. (2020) 1(8):443–9. 10.1302/2633-1462.18.bjo-2020-0089.r1 33215137 PMC7667224

[40] Mau M Thorne T Rossbach K Sato EH Zhang C Presson AP. Does weightbearing status impact mortality and other complications in hip fracture patients when accounting for frailty? Bone Joint Open. (2025) 6(7):785–95. 10.1302/2633-1462.67.BJO-2025-0059.R1 40625097 PMC12235488

[41] Palaniappan S Soiza RL Duffy S Moug SJ Myint PK. Comparison of the clinical frailty score (CFS) to the national emergency laparotomy audit (NELA) risk calculator in all patients undergoing emergency laparotomy. Colorectal Dis. (2022) 24(6):782–9. 10.1111/codi.16089 35167177 PMC9311201

[42] Grudzinski AL Aucoin S Talarico R Moloo H Lalu MM McIsaac DI. Comparing the predictive accuracy of frailty instruments applied to preoperative electronic health data for adults undergoing noncardiac surgery. Br J Anaesth. (2022) 129(4):506–14. 10.1016/j.bja.2022.07.019 36031416

[43] Man J Xu J. The administrative risk analysis index (RAI-A) does not predict reintervention or mortality within 30 days in patients who undergo elective open or endovascular abdominal aortic aneurysm repair. J Surg Res. (2021) 264:179–85. 10.1016/j.jss.2021.02.015 33836407

[44] Spitzer H Yang R Bohan PK Chang S-C Grunkemeier G Vreeland T. Preoperative risk prediction for pancreatectomy: a comparative analysis of three scoring systems. J Surg Res. (2022) 279:374–82. 10.1016/j.jss.2022.06.033 35820319

[45] McQuestion CC Ballacchino MM Gershgorn JI Gajdos C Dosluoglu HH Nader ND. The validity of frailty scoring in preoperative risk assessment for patients in the surgical intensive care unit. J Surg Res. (2026) 317:277–88. 10.1016/j.jss.2025.11.001 41389381

[46] Deol ES Sharma V Fadel AE Vasdev R Henning G Basourakos S. Comparing frailty indices for risk stratification in urologic oncology: which index to choose? Urology. (2024) 194:154–61. 10.1016/j.urology.2024.08.055 39214499

[47] Ehlert BA Najafian A Orion KC Malas MB Black JH III Abularrage CJ. Validation of a modified frailty index to predict mortality in vascular surgery patients. J Vasc Surg. (2016) 63(6):1595–1601.e2. 10.1016/j.jvs.2015.12.023 26926932

[48] Gaston BT Bellomo TR Salomon B Mulaney-Topkar B Demsas F Anjorin A. Direct comparison of frailty scores and their association with postoperative outcomes in patients undergoing lower extremity revascularization. J Vasc Surg. (2026) 83(2):539–53. 10.1016/j.jvs.2025.09.033 40976447

[49] Zamanzadeh RS Seilern und Aspang JRM Schwartz AM Martin JR Premkumar A Wilson JM. Age-adjusted modified frailty index predicts 30-day complications and mortality in aseptic revision total hip and knee arthroplasty. J Arthroplasty. (2024) 39(1):198–205. 10.1016/j.arth.2023.06.032 37380143

[50] Pean CA Buddhiraju A Shimizu MR Chen TLW Esposito JG Kwon YM. Prediction of 30-day mortality following revision total hip and knee arthroplasty: machine learning algorithms outperform CARDE-B, 5-item, and 6-item modified frailty index risk scores. J Arthroplasty. (2024) 39(11):2824–30. 10.1016/j.arth.2024.05.056 38797444

[51] Farid M Wang J Perdikis B Blasdel N Darko K O’Leary S. Assessing the assessors: comparative performance of frailty and nutritional metrics in predicting 30-day morbidity and mortality after cranial neurosurgery. J Clin Neurosci. (2026) 150:112047. 10.1016/j.jocn.2026.112047 42068839

[52] Sabet CJ Jain B Lawand J Prulovic S Young B Nguyen D. Comparative performance of the risk analysis index versus traditional frailty measures in predicting outcomes following unicompartmental knee arthroplasty: a national database analysis. Knee. (2026) 58:104261. 10.1016/j.knee.2025.10.015 41219082

[53] Maegawa FB Ahmad M Aguirre K Elhanafi S Chiba S Philipovskiy A. The impact of minimally invasive surgery and frailty on post-hepatectomy outcomes. HPB (Oxford). (2022) 24(9):1577–84. 10.1016/j.hpb.2022.03.018 35459620

[54] Yang Y-F Zhang P Wu B Wang S-Y Guo H-W Zheng Q-X. Preoperative frailty as a key predictor of short- and long-term outcomes among octogenarians undergoing hepatectomy for hepatocellular carcinoma: a multicenter comprehensive analysis. HPB (Oxford). (2024) 26(12):1495–504. 10.1016/j.hpb.2024.08.010 39271376

[55] Owodunni OP Yocky AG Courville EN Peter-Okaka U Alare KP Schmidt M. A comprehensive analysis of the triad of frailty, aging, and obesity in spine surgery: the risk analysis index predicted 30-day mortality with superior discrimination. Spine J. (2023) 23(12):1778–89. 10.1016/j.spinee.2023.08.008 37625550

[56] Lima DFT Cristelo D Reis P Abelha F Mourão J. Outcome prediction with physiological and operative severity score for the enumeration of mortality and morbidity score system in elderly patients submitted to elective surgery. Saudi J Anaesth. (2019) 13(1):46–51. 10.4103/sja.sja_206_18 30692888 PMC6329253

[57] Warrier A Kim N Bartholomew R Benchetrit L Lee DJ. Otologic surgery risk prediction: risk analysis index-administrative versus modified frailty index-5. Laryngoscope. (2026) 136(9):3955–65. 10.1002/lary.70539 41928719 PMC13460496

[58] Isand KG Hussain S Sadiqi M Kirsimägi Ü Bond-Smith G Kolk H. Frailty assessment can enhance current risk prediction tools in emergency laparotomy: a retrospective cohort study. World J Surg. (2023) 47(11):2688–97. 10.1007/s00268-023-07140-z 37589793

[59] McIsaac DI Aucoin SD Van Walraven C. A Bayesian comparison of frailty instruments in noncardiac surgery: a cohort study. Anesth Analg. (2021) 133(2):366–73. 10.1213/ane.0000000000005290 33264118

[60] Allen MB Orkaby AR Justice S Hall DE Hu FY Cooper Z. Frailty and outcomes following cardiopulmonary resuscitation for perioperative cardiac arrest. JAMA Netw Open. (2023) 6(7):e2321465. 10.1001/jamanetworkopen.2023.21465 37399014 PMC10318473

[61] Zaib J Madni A Saad Azhar M. Predictive value of comprehensive geriatric assessment scores for mortality in patients with hip fracture: a retrospective cohort study. Cureus. (2023) 15(9):e45070. 10.7759/cureus.45070 37842357 PMC10568117

[62] Cartagena-Reyes MA Solomon E Silva Aponte J Joshi A Raad M Hassanzadeh H. Development of a novel risk stratification score to predict 30-day mortality in cervical trauma patients. Clin Spine Surg. (2024) 37(6):275–81. 10.1097/bsd.0000000000001596 38490969

[63] Moya AN Owodunni OP Harrison JL Shahriari SR Shetty AK Borah GL. Preoperative frailty risk in cranioplasty patients: risk analysis index predicts adverse outcomes. PlastReconstr Surg Glob Open. (2023) 11(6):e5059. 10.1097/gox.0000000000005059 PMC10284328 37351116

[64] Gupta NK Chmait HR Gill V Turnow M Manes T Taylor BC. Risk analysis index for estimation of 30-day postoperative mortality in hip fractures. JAMA Netw Open. (2025) 8(5):e2512689. 10.1001/jamanetworkopen.2025.12689 40440016 PMC12123473

[65] Aakre EK Aakre KM Flaatten H Hufthammer KO Ranhoff AH Jammer I. High-sensitivity cardiac troponin T and frailty predict short-term mortality in patients ≥75 years undergoing emergency abdominal surgery: a prospective observational study. Anesth Analg. (2024) 139(2):313–22. 10.1213/ane.0000000000006845 39008976

[66] Courville E Rumalla K Kazim SF Dicpinigaitis AJ Schmidt M Robinson TM. Risk analysis index as a preoperative frailty tool for elective ventriculoperitoneal shunt surgery for idiopathic normal pressure hydrocephalus. J Neurosurg. (2024) 140(4):1110–6. 10.3171/2023.7.jns23767 38564806

[67] Bowers CA Varela S Conlon M Kazim SF Thommen R Roster K. Comparison of the risk analysis index and the modified 5-factor frailty index in predicting 30-day morbidity and mortality after spine surgery. J Neurosurg Spine. (2023) 39(1):136–45. 10.3171/2023.2.spine221019 37029672

[68] Bowers CA Varela S Naftchi AF Kazim SF Hall DE Ng C. Superior discrimination of the risk analysis index compared with the 5-item modified frailty index in 30-day outcome prediction after anterior cervical discectomy and fusion. J Neurosurg Spine. (2023) 39(4):509–19. 10.3171/2023.5.spine221020 37439459

[69] Soni C Koltenyuk V Gupta N Arif HA Areti A Manes T. Utility of revised risk analysis index as a predictor of mortality and morbidity in orthopaedic trauma. J Am Acad Orthop Surg Glob Res Rev. (2025) 9(6):e25.00086. 10.5435/jaaosglobal-d-25-00086 40493046 PMC12142732

[70] Elsamadicy AA Serrato P Ghanekar SD Hansen J Brown EDL Khalid SI. Composite RAI, malnutrition, and anemia model superiorly predicts 30-day morbidity and mortality after surgery for adult spinal deformity. J Clin Med. (2025) 14(15):5379. 10.3390/jcm14155379 40807001 PMC12347132

[71] Williamson TK Aziz A Bluhm B McDonald C Verlinsky L Checketts J. Does pre-operative metabolic and nutritional assessment have greater association with perioperative complications than frailty following surgical intervention for proximal humerus fractures? JSES Rev Rep Tech. (2026) 6(2):100686. 10.1016/j.xrrt.2026.100686 41858490 PMC12995877

[72] Paiz CC Owodunni OP Courville EN Schmidt M Alunday R Bowers CA. Frailty predicts 30-day mortality following major complications in neurosurgery patients: the risk analysis index has superior discrimination compared to modified frailty index-5 and increasing patient age. World Neurosurg X. (2024) 23:100286. 10.1016/j.wnsx.2024.100286 38516023 PMC10955078

[73] Hermann CE Koelper NC Andriani L Latif NA Ko EM. Predictive value of 5-factor modified frailty index in oncologic and benign hysterectomies. Gynecol Oncol Rep. (2022) 43:101063. 10.1016/j.gore.2022.101063 36051500 PMC9424918

[74] Grudzinski AL Aucoin S Talarico R Moloo H Lalu MM McIsaac DI. Measuring the predictive accuracy of preoperative clinical frailty instruments applied to electronic health data in older patients having emergency general surgery. Ann Surg. (2023) 278(2):e341–8. 10.1097/sla.0000000000005718 36134577

[75] Yocky AG Owodunni OP Courville EN Kazim SF Schmidt MH Gearhart SL. The risk analysis index has superior discrimination compared with the modified frailty index-5 in predicting worse postoperative outcomes for the octogenarian neurosurgical patient. Neurosurg Pract. (2023) 4(3):e00044. 10.1227/neuprac.0000000000000044 39958794 PMC11809970

[76] Skandalakis GP Medani K Rumalla K Roy JM Segura A Zohdy YM. Preoperative frailty and 30-day mortality after resection of brain metastases: a multicenter surgical registry analysis of 11,038 patients. Neurosurg Focus. (2023) 55(2):E8. 10.3171/2023.5.focus23198 37527672

[77] Hall DE Arya S Schmid KK Carlson MA Lavedan P Bailey TL. Association of a frailty screening initiative with postoperative survival at 30, 180, and 365 days. JAMA Surg. (2017) 152(3):233–40. 10.1001/jamasurg.2016.4219 27902826 PMC7180387

[78] Rothenberg KA George EL Trickey AW Barreto NB Johnson TM II Hall DE. Assessment of the risk analysis index for prediction of mortality, major complications, and length of stay in patients who underwent vascular surgery. Ann Vasc Surg. (2020) 66:442–53. 10.1016/j.avsg.2020.01.015 31935435 PMC7608558

[79] Kazim SF Dicpinigaitis AJ Bowers CA Shah S Couldwell WT Thommen R. Frailty status is a more robust predictor than age of spinal tumor surgery outcomes: a NSQIP analysis of 4,662 patients. Neurospine. (2022) 19(1):53–62. 10.14245/ns.2142770.385 35130424 PMC8987561

[80] Conlon M Thommen R Kazim SF Dicpinigaitis AJ Schmidt MH McKee RG. Risk analysis index and its recalibrated version predict postoperative outcomes better than 5-factor modified frailty index in traumatic spinal injury. Neurospine. (2022) 19(4):1039–48. 10.14245/ns.2244326.163 36597640 PMC9816576

[81] Arteaga AS Aguilar LT González JT Boza AS Muñoz-Cruzado VD Ciuró FP. Impact of frailty in surgical emergencies. A comparison of four frailty scales. Eur J Trauma Emerg Surg. (2021) 47(5):1613–9. 10.1007/s00068-020-01314-3 32036392

[82] McConaghy KM Orr MN Emara AK Sinclair ST Klika AK Piuzzi NS. Can extant comorbidity indices identify patients who experience poor outcomes following total joint arthroplasty? Arch Orthop Trauma Surg. (2023) 143(3):1253–63. 10.1007/s00402-021-04250-y 34787694

[83] Musharbash FN Khalifeh JM Raad M Puvanesarajah V Lee SH Neuman BJ. Predicting 30-day mortality after surgery for metastatic disease of the spine: the H2-FAILS score. Eur Spine J. (2023) 32(7):2513–20. 10.1007/s00586-023-07713-5 37186159

[84] Prvulovic S Gupta N Zhogi S Bowers C Shah K Marcinkowski A. Predictive value of the risk analysis index for 30-day mortality following surgical management of thoracolumbar vertebral body fractures. Eur Spine J. (2026) 35:1964–74. 10.1007/s00586-025-09289-8 40853456

[85] Gupta N Sbaih O DiCiurcio W III Miller M Nanavati R Smith H. ACDF and cervical disc replacement for single-level cervical spine degenerative disease: a frailty driven propensity score-matched comparative analysis. Eur Spine J. (2026) 35(9):5362–75. 10.1007/s00586-026-09780-w 41636867

[86] Leigh N Williams GA Strasberg SM Fields RC Hawkins WG Hammill CW. Increased morbidity and mortality after hepatectomy for colorectal liver metastases in frail patients is largely driven by worse outcomes after minor hepatectomy: it’s not “just a wedge”. Ann Surg Oncol. (2022) 29(9):5476–85. 10.1245/s10434-022-11830-8 35595939

[87] Elsamadicy AA Serrato P Sadeghzadeh S Sayeed S Hengartner AC Khalid SI. Assessing a revised-risk analysis index for morbidity and mortality after spine surgery for metastatic spinal tumors. J Neuro Oncol. (2025) 171(1):213–28. 10.1007/s11060-024-04830-z 39320656

[88] Elsamadicy AA Serrato P Ghanekar SD Brown EDL Ward M Pennington Z. Assessing combined effects of risk analysis index-revised (RAI-rev), malnutrition, and anemia on morbidity and mortality after spine surgery for metastatic spinal tumors. J Neuro Oncol. (2025) 174(2):527–37. 10.1007/s11060-025-05071-4 40405043

[89] Hinojosa-Gonzalez DE Saffati G Salgado-Garza G Armengol Garcia C Chaput M Yu B. Frailty index and nutrition status impact perioperative outcomes in patients undergoing minimally invasive radical nephrectomy. J Robot Surg. (2025) 19(1):147. 10.1007/s11701-025-02305-5 40214944

[90] Darvall JN Loth J Bose T Braat S De Silva A Story DA. Accuracy of the clinical frailty scale for perioperative frailty screening: a prospective observational study. Can J Anesth. (2020) 67(6):694–705. 10.1007/s12630-020-01610-x 32128722

[91] Snitkjær C Rehné Jensen L í Soylu L Hauge C Kvist M Jensen TK. Impact of clinical frailty on surgical and non-surgical complications after major emergency abdominal surgery. BJS Open. (2024) 8(3):zrae039. 10.1093/bjsopen/zrae039 PMC11126315 38788680

[92] Diao Y-K Li D Wu H Yang Y-F Wang N-Y Gu W-M. Association of preoperative frailty with short- and long-term outcomes after hepatic resection for elderly patients with hepatocellular carcinoma: multicentre analysis. BJS Open. (2025) 9(1):zrae171. 10.1093/bjsopen/zrae171 PMC11806262 39921532

[93] George EL Jacobs MA Reitz KM Massarweh NN Youk AO Arya S. Outcomes of women undergoing noncardiac surgery in veterans affairs compared with non–veterans affairs care settings. JAMA Surg. (2024) 159(5):501–9. 10.1001/jamasurg.2023.8081 38416481 PMC10902781

[94] Le ST Liu VX Kipnis P Zhang J Peng PD Cespedes Feliciano EM. Comparison of electronic frailty metrics for prediction of adverse outcomes of abdominal surgery. JAMA Surg. (2022) 157(5):e220172. 10.1001/jamasurg.2022.0172 35293969 PMC8928095

[95] George EL Hall DE Youk A Chen R Kashikar A Trickey AW. Association between patient frailty and postoperative mortality across multiple noncardiac surgical specialties. JAMA Surg. (2021) 156(1):e205152. 10.1001/jamasurg.2020.5152 33206156 PMC7675216

[96] Shinall MC Arya S Youk A Varley P Shah R Massarweh NN. Association of preoperative patient frailty and operative stress with postoperative mortality. JAMA Surg. (2020) 155(1):e194620. 10.1001/jamasurg.2019.4620 31721994 PMC6865246

