# Supplementary material for: Modified frailty index versus risk analysis index for 30-day mortality after noncardiac surgery: a systematic review and meta-analysis
Source: Front Med (Lausanne). 2026 Oct 9;13:1885620. doi: 10.3389/fmed.2026.1885620 (PMC13690649; doi:10.3389/fmed.2026.1885620)
Supplement: Supplementary file 1 [file Datasheet1.pdf]

Supplementary Appendix 1. Search Strategies and Retrieval Counts

Study title: Comparative Prognostic Discrimination of the Modified Frailty Index and Risk Analysis Index for 30-Day Mortality After Noncardiac Surgery: A Systematic Review and Meta-analysis

PROSPERO registration: CRD420261378778 (initial registration April 25, 2026; amended May 12, 2026)

Date of search: 11 May 2026

Date range: From database inception to 11 May 2026

Languages: No date restrictions were applied. Searches were not restricted by language at the database level; however, inclusion required sufficient information for eligibility assessment and data extraction.

Databases searched: PubMed (MEDLINE), Embase (Elsevier), Scopus, Web of Science Core Collection, Cochrane Central Register of Controlled Trials (CENTRAL), China National Knowledge Infrastructure (CNKI). ClinicalTrials.gov was searched as an additional source to identify potentially relevant ongoing or unpublished records.

Number of records retrieved per database is reported in the PRISMA 2020 flow diagram (Figure 1 of the main manuscript).

==

1. PubMed (MEDLINE)

Search interface: https://pubmed.ncbi.nlm.nih.gov/ Records retrieved: 3,663

Search string:

(frailty[Title/Abstract] OR frail[Title/Abstract] OR "Clinical Frailty Scale"[Title/Abstract] OR CFS[Title/Abstract] OR "modified Frailty Index"[Title/Abstract] OR "modified frailty index"[Title/Abstract] OR mFI[Title/Abstract] OR mFI-5[Title/Abstract] OR mFI-11[Title/Abstract] OR "Risk Analysis Index"[Title/Abstract] OR RAI[Title/Abstract] OR "Edmonton Frail Scale"[Title/Abstract] OR EFS[Title/Abstract] OR "Fried phenotype"[Title/Abstract] OR "Fried frailty phenotype"[Title/Abstract] OR "FRAIL scale"[Title/Abstract]) AND (surg*[Title/Abstract] OR operat*[Title/Abstract] OR preoperat*[Title/Abstract] OR perioperat*[Title/Abstract] OR postoperat*[Title/Abstract]) AND (predict*[Title/Abstract] OR prognos*[Title/Abstract] OR discriminat*[Title/Abstract] OR "predictive performance"[Title/Abstract] OR "prognostic performance"[Title/Abstract] OR "risk assessment"[Title/Abstract] OR "risk stratification"[Title/Abstract] OR AUC[Title/Abstract] OR "c-statistic"[Title/Abstract] OR "C statistic"[Title/Abstract] OR "area under the curve"[Title/Abstract] OR ROC[Title/Abstract] OR "receiver operating

characteristic"[Title/Abstract]) AND (mortality[Title/Abstract] OR death[Title/Abstract] OR delirium[Title/Abstract] OR complication*[Title/Abstract] OR readmission[Title/Abstract] OR "length of stay"[Title/Abstract] OR discharge[Title/Abstract] OR "non-home discharge"[Title/Abstract]) NOT ("cardiac surgery"[Title/Abstract] OR CABG[Title/Abstract] OR "coronary artery bypass"[Title/Abstract] OR "valve surgery"[Title/Abstract] OR TAVI[Title/Abstract] OR TAVR[Title/Abstract] OR "cardiopulmonary bypass"[Title/Abstract] OR polytrauma[Title/Abstract] OR "major trauma"[Title/Abstract] OR burn*[Title/Abstract])

==

2. Embase (Elsevier)

Search interface: https://www.embase.com/ Records retrieved: 2,475

Search string:

('clinical frailty scale':ti,ab,kw OR 'modified frailty index':ti,ab,kw OR 'mfi-5':ti,ab,kw OR 'mfi-11':ti,ab,kw OR 'risk analysis index':ti,ab,kw OR 'edmonton frail scale':ti,ab,kw OR 'fried phenotype':ti,ab,kw OR 'fried frailty phenotype':ti,ab,kw OR 'frail scale':ti,ab,kw) AND (surgery:ti,ab,kw OR surgical:ti,ab,kw OR preoperative:ti,ab,kw OR perioperative:ti,ab,kw OR postoperative:ti,ab,kw OR orthopedic:ti,ab,kw OR orthopaedic:ti,ab,kw OR abdominal:ti,ab,kw OR colorectal:ti,ab,kw OR gastrointestinal:ti,ab,kw OR thoracic:ti,ab,kw OR urologic:ti,ab,kw OR vascular:ti,ab,kw OR 'hip fracture':ti,ab,kw) AND (mortality:ti,ab,kw OR death:ti,ab,kw OR delirium:ti,ab,kw OR complication*:ti,ab,kw OR morbidity:ti,ab,kw OR readmission:ti,ab,kw OR 'length of stay':ti,ab,kw OR discharge:ti,ab,kw OR 'non-home discharge':ti,ab,kw) NOT ('cardiac surgery':ti,ab,kw OR cabg:ti,ab,kw OR 'coronary artery bypass':ti,ab,kw OR 'valve surgery':ti,ab,kw OR tavi:ti,ab,kw OR tavr:ti,ab,kw OR 'cardiopulmonary bypass':ti,ab,kw OR polytrauma:ti,ab,kw OR 'major trauma':ti,ab,kw OR burn*:ti,ab,kw)

==

3. Scopus

Search interface: https://www.scopus.com/ Records retrieved: 2,127

Search string:

TITLE-ABS-KEY("Clinical Frailty Scale" OR "modified Frailty Index" OR "modified frailty index" OR "mFI-5" OR "mFI-11" OR "Risk Analysis Index" OR "Edmonton Frail Scale" OR "Fried phenotype" OR "Fried frailty phenotype" OR "FRAIL scale") AND TITLE-ABS-KEY(surgery OR surgical OR preoperative OR perioperative OR postoperative OR orthopedic OR orthopaedic OR abdominal OR colorectal OR gastrointestinal OR thoracic OR urologic OR vascular OR "hip fracture") AND TITLE-ABS-KEY(mortality OR death OR delirium OR complication* OR morbidity OR readmission OR "length of stay" OR discharge

OR "non-home discharge") AND NOT TITLE-ABS-KEY("cardiac surgery" OR CABG OR "coronary artery bypass" OR "valve surgery" OR TAVI OR TAVR OR "cardiopulmonary bypass" OR polytrauma OR "major trauma" OR burn*)

===

4. Web of Science Core Collection

Search interface: https://www.webofscience.com/ Records retrieved: 3,239

Search string:

TS=(("Clinical Frailty Scale" OR "modified Frailty Index" OR "modified frailty index" OR "mFI-5" OR "mFI-11" OR "Risk Analysis Index" OR "Edmonton Frail Scale" OR "Fried phenotype" OR "Fried frailty phenotype" OR "FRAIL scale") OR ((frailty OR frail) NEAR/3 (scale OR index OR score OR screening OR assessment OR tool OR phenotype))) AND TS=(surgery OR surgical OR preoperative OR perioperative OR postoperative OR orthopedic OR orthopaedic OR abdominal OR colorectal OR gastrointestinal OR thoracic OR urologic OR vascular OR "hip fracture") AND TS=(mortality OR death OR delirium OR complication* OR morbidity OR readmission OR "length of stay" OR discharge OR "non-home discharge") NOT TS=("cardiac surgery" OR CABG OR "coronary artery bypass" OR "valve surgery" OR TAVI OR TAVR OR "cardiopulmonary bypass" OR polytrauma OR "major trauma" OR burn*)

===

5. Cochrane Central Register of Controlled Trials (CENTRAL)

Search interface: https://www.cochranelibrary.com/ Records retrieved: 111

Search string:

("Clinical Frailty Scale" OR "modified Frailty Index" OR "mFI-5" OR "mFI-11" OR "Risk Analysis Index" OR "Edmonton Frail Scale" OR "Fried phenotype" OR "FRAIL scale"):ti,ab,kw AND (surgery OR surgical OR preoperative OR perioperative OR postoperative OR orthopedic OR orthopaedic OR abdominal OR colorectal OR gastrointestinal OR thoracic OR urologic OR vascular OR "hip fracture"):ti,ab,kw AND (mortality OR death OR delirium OR complications OR morbidity OR readmission OR "length of stay" OR discharge OR "non-home discharge"):ti,ab,kw

===

6. China National Knowledge Infrastructure (CNKI)

Search interface: https://www.cnki.net/ Records retrieved: 36

Search string (original Chinese):

主题 =(临床衰弱量表 OR 临床虚弱量表 OR 改良衰弱指数 OR 改良虚弱指数 OR 风险分析指数 OR 埃德蒙顿衰弱量表 OR 埃德蒙顿虚弱量表 OR Fried 表型 OR Fried 衰弱表型 OR FRAIL 量表 OR Clinical Frailty Scale OR modified Frailty Index OR Risk Analysis Index OR Edmonton Frail Scale OR Fried phenotype OR FRAIL scale) AND 主题 =(手术 OR 外科 OR 围术期 OR 术前 OR 术后 OR 骨科 OR 腹部手术 OR 胃肠外科 OR 结直肠手术 OR 胸外科 OR 泌尿外科 OR 血管外科 OR 髋部骨折) AND 主题 =(死亡 OR 谵妄 OR 并发症 OR 再入院 OR 住院时间 OR 出院去向 OR 非居家出院 OR 预后) NOT 主题 =(心脏手术 OR 心脏外科 OR 冠状动脉旁路移植 OR 搭桥 OR 瓣膜手术 OR 多发伤 OR 严重创伤 OR 烧伤)

English back-translation:

Topic = (Clinical Frailty Scale [临床衰弱量表 / 临床虚弱量表] OR modified Frailty Index [改良衰弱指数 / 改良虚弱指数] OR Risk Analysis Index [风险分析指数] OR Edmonton Frail Scale [埃德蒙顿衰弱量表 / 埃德蒙顿虚弱量表] OR Fried phenotype [Fried 表型 / Fried 衰弱表型] OR FRAIL scale [FRAIL 量表] OR English-language equivalents) AND Topic = (surgery [手术] OR surgical/surgery department [外科] OR perioperative [围术期] OR preoperative [术前] OR postoperative [术后] OR orthopedics [骨科] OR abdominal surgery [腹部手术] OR gastrointestinal surgery [胃肠外科] OR colorectal surgery [结直肠手术] OR thoracic surgery [胸外科] OR urological surgery [泌尿外科] OR vascular surgery [血管外科] OR hip fracture [髋部骨折]) AND Topic = (death [死亡] OR delirium [谵妄] OR complications [并发症] OR readmission [再入院] OR length of stay [住院时间] OR discharge destination [出院去向] OR non-home discharge [非居家出院] OR prognosis [预后]) NOT Topic = (cardiac surgery [心脏手术 / 心脏外科] OR coronary artery bypass grafting [冠状动脉旁路移植] OR bypass [搭桥] OR valve surgery [瓣膜手术] OR polytrauma [多发伤] OR severe trauma [严重创伤] OR burns [烧伤])

==

7. ClinicalTrials.gov (additional source for ongoing/unpublished records)

Search interface: https://clinicaltrials.gov/ Records retrieved: 183

Search terms: frailty AND Surgery

==

Summary of records retrieved

Database — Records

PubMed (MEDLINE) — 3,663

Embase — 2,475

Scopus — 2,127

Web of Science Core Collection — 3,239

Cochrane CENTRAL — 111

China National Knowledge Infrastructure (CNKI) — 36

Subtotal (bibliographic databases) — 11,651

ClinicalTrials.gov (additional source) — 183

Total records before deduplication — 11,834

==

End of Supplementary Appendix 1

Supplementary Appendix 2. Risk of Bias Assessment of Included Studies Using the Quality In Prognosis Studies (QUIPS) Tool

First author, year	Journal	Study design	Data source / database	Surgical population	Frailty tool (s)	Domain 1: Study Participation	Domain 2: Study Attrition	Domain 3: Prognostic Factor Measurement	Domain 4: Outcome Measurement	Domain 5: Study Confounding	Domain 6: Statistical Analysis and Reporting	Overall risk of bias	Brief justification
Aakre E 2024	Anesthesia and Analgesia	prospective observational cohort (biomarker substudy of ProPEL trial)	single-center prospective (Haukeland University Hospital, Norway)	Adults >=75 undergoing emergency abdominal surgery (intraperitoneal, within 72 hours), vascular emergency and palliative surgery excluded; n=146	CFS	Low	Moderate	Low	Low	Low	Moderate	Moderate	Two Moderate ratings (D2: incomplete blood sample ascertainment from screened cohort; D6: bootstrap-estimated AUC with threshold uncertainty) with no High domains; 2 Moderate = Moderate overall RoB per rubric.
Allen M 2023	JAMA Network Open	retrospective registry-based cohort	ACS-NSQIP 2015-2020	Adults >=50 undergoing non-cardiac surgery who received CPR on postoperative day 0 (perioperative	RAI	Moderate	Low	Moderate	Low	Moderate	High	High	Domain 6 rated High (no AUC/c-statistic for 30-day mortality) triggers overall High risk of bias.

First author, year	Journal	Study design	Data source / database	Surgical population	Frailty tool (s)	Domain 1: Study Participation	Domain 2: Study Attrition	Domain 3: Prognostic Factor Measurement	Domain 4: Outcome Measurement	Domain 5: Study Confounding	Domain 6: Statistical Analysis and Reporting	Overall risk of bias	Brief justification
				cardiac arrest), n=3058, ACS-NSQIP 2015-2020									
Arya 2020	Annals of Surgery	prospective cohort	VASQIP; ACS-NSQIP; single-center VA prospective	Adults undergoing major elective non-cardiac surgery; VASQIP (2010-2014, n=480,731), ACS-NSQIP external validation (2005-2014, n=1,391,785), and prospective cohort at NWIHCS VA (n=6,856); mixed surgical specialties	RAI	Low	Low	Low	Low	Low	Low	Low	All 6 domains rated Low → Overall Low per rubric.
Bowers C	Journal	retrospectiv	ACS-NSQIP	Adults >18	mFI +	Low	Low	Moder	Low	Low	Low	Mod	One Moderate at

First author, year	Journal	Study design	Data source / database	Surgical population	Frailty tool (s)	Domain 1: Study Participation	Domain 2: Study Attrition	Domain 3: Prognostic Factor Measurement	Domain 4: Outcome Measurement	Domain 5: Study Confounding	Domain 6: Statistical Analysis and Reporting	Overall risk of bias	Brief justification
2023	of Neurosurgery: Spine	e registry-based cohort (longitudinal)	2015-2019	undergoing spine surgery; ACS-NSQIP 2015-2019; n=292,225 overall; subgroups: infection (n=3666), trauma (n=6693), tumor (n=6391)	RAI			ate				erate	Domain 3 (NSQIP-derived administrative RAI without paper-specific validation, with some variables excluded) with no High domains; one Moderate at D3 = Moderate overall RoB per rubric.
Bowers C 2023	Journal of Neurosurgery: Spine	retrospective registry-based cohort	ACS-NSQIP 2015-2019	Adults >18 undergoing ACDF for degenerative spinal disease	mFI + RAI	Low	Low	Moderate	Low	Low	Low	Moderate	D3 is Moderate (NSQIP-derived RAI-rev without in-paper validation); any Moderate at D3 yields overall Moderate by rubric rule.
Branstetter RM IV 2024	World Neurosurgery	retrospective registry-bas	ACS-NSQIP 2012–2020	Overweight or obese adults (BMI ≥25)	mFI + RAI	Low	Low	Moderate	Low	Low	Low	Moderate	One domain (D3) is Moderate due to registry-derived

First author, year	Journal	Study design	Data source / database	Surgical population	Frailty tool (s)	Domain 1: Study Participation	Domain 2: Study Attrition	Domain 3: Prognostic Factor Measurement	Domain 4: Outcome Measurement	Domain 5: Study Confounding	Domain 6: Statistical Analysis and Reporting	Overall risk of bias	Brief justification
		ed cohort		undergoing neurosurgery procedures (spine, skull base, vascular/functional)									tool without in-study validation; single Moderate at D3 (high-weight domain) triggers Moderate overall per the rubric.
Cartagena-Reyes M 2024	Clinical Spine Surgery	retrospective registry-based cohort with internal and external validation	ACS-NSQIP 2012-2020 (primary); NIS 2012-2014 (external validation); TQIP 2007-2016 (benchmark comparison)	Adults undergoing instrumented cervical spine surgery for traumatic injury; ACS-NSQIP 2012-2020; n=822	mFI	Moderate	Low	Moderate	Low	Low	Moderate	Moderate	Three Moderate domains (D1, D3, D6) with no High; 2-3 Moderate = Moderate overall RoB per rubric.
Chen X 2024	BMC Geriatrics	retrospective cohort	single-center (First Affiliated Hospital of Anhui Medical	Elderly esophageal cancer patients (age >=65) undergoing radical surgery,	mFI	Low	Low	Moderate	Moderate	Moderate	High	High	Domain 6 rated High (no AUC for 30-day mortality) triggers overall High risk of bias regardless of other

First author, year	Journal	Study design	Data source / database	Surgical population	Frailty tool (s)	Domain 1: Study Participation	Domain 2: Study Attrition	Domain 3: Prognostic Factor Measurement	Domain 4: Outcome Measurement	Domain 5: Study Confounding	Domain 6: Statistical Analysis and Reporting	Overall risk of bias	Brief justification
			University, China)	n=515, single-center China, 2021									domain ratings.
Conlon M 2022	Neurospine	retrospective registry-based cohort	ACS-NSQIP 2015-2019	Adults ≥18 undergoing surgical intervention for traumatic spinal injuries (TSI)	mFI + RAI	Moderate	Low	Moderate	Low	Moderate	Low	Moderate	D3 is Moderate (registry-derived RAI with excluded cognitive items), D1 and D5 are also Moderate; 3 Moderate domains with no High → overall Moderate.
Courville E 2024	Journal of Neurosurgery	retrospective registry-based cohort (population-based longitudinal analysis)	Nationwide Readmissions Database (NRD) 2018-2019	Adults >=60 with idiopathic normal pressure hydrocephalus undergoing elective VPS surgery; NRD 2018-2019; n=9319	mFI + RAI	Moderate	Moderate	Moderate	Moderate	Moderate	Low	High	Four Moderate domains (D1, D2, D3, D4) with no High; four or more Moderate domains = High overall RoB per rubric.
Darvall J 2020	Canadian Journal of	prospective single-centre	Royal Melbourne Hospital,	Adults >=65 years undergoing elective or	mFI	Moderate	Low	Low	Moderate	Low	High	High	Domain 6 rated High (standalone tool AUC for

First author, year	Journal	Study design	Data source / database	Surgical population	Frailty tool (s)	Domain 1: Study Participation	Domain 2: Study Attrition	Domain 3: Prognostic Factor Measurement	Domain 4: Outcome Measurement	Domain 5: Study Confounding	Domain 6: Statistical Analysis and Reporting	Overall risk of bias	Brief justification
	Anaesthesia	observational cohort	Australia (single-centre, 2017-2018)	emergency non-endoscopy surgery at Royal Melbourne Hospital (n=218)									30-day mortality cannot be extracted as no mortality ROC analysis was performed); any High = High overall.
Deol E 2024	Urology	retrospective registry-based cohort	ACS-NSQIP 2013-2017	Adults >=18 undergoing radical prostatectomy, partial/radical nephrectomy, or radical cystectomy; NSQIP 2013-2017; n=101,739	mFI + RAI	Low	Low	Moderate	Low	Low	Low	Moderate	One Moderate at D3 (high-weight domain) with all other domains Low triggers Moderate overall per rule.
Diao Y 2025	BJS Open	multicentre retrospective cohort	Ten Chinese hepatobiliary centres, 2012-2021	Elderly patients (>=70 years) with HCC undergoing curative-intent hepatic resection	mFI	Low	Moderate	Low	Moderate	Low	High	High	Domain 6 rated High (no AUC/c-statistic for 30-day mortality or any survival endpoint); any High

First author, year	Journal	Study design	Data source / database	Surgical population	Frailty tool (s)	Domain 1: Study Participation	Domain 2: Study Attrition	Domain 3: Prognostic Factor Measurement	Domain 4: Outcome Measurement	Domain 5: Study Confounding	Domain 6: Statistical Analysis and Reporting	Overall risk of bias	Brief justification
				at 10 Chinese hospitals (n=488)									= High overall.
Ehlert B 2016	Journal of Vascular Surgery	retrospective registry-based cohort	ACS-NSQIP 2006-2012	Adults undergoing carotid revascularization, AAA repair, or lower extremity revascularization for PAD; NSQIP 2006-2012; n=72,106; emergency cases excluded	mFI	Moderate	Low	Moderate	Low	Moderate	Low	Moderate	D1=Moderate, D3=Moderate (high-weight), D5=Moderate gives 3 Moderates with no High; per rule 2-3 Moderates = Moderate overall.
Elsamadicy A 2025	Journal of Neuro-Oncology	retrospective registry-based cohort	ACS-NSQIP 2011-2022	Adults undergoing laminectomy or corpectomy (+-fusion) for extradural metastatic spinal tumors (n=1,530)	RAI	Low	Low	Moderate	Low	Low	Moderate	Moderate	Two Moderate ratings (D3 and D6) with no High; 2-3 Moderate = Moderate overall.

First author, year	Journal	Study design	Data source / database	Surgical population	Frailty tool (s)	Domain 1: Study Participation	Domain 2: Study Attrition	Domain 3: Prognostic Factor Measurement	Domain 4: Outcome Measurement	Domain 5: Study Confounding	Domain 6: Statistical Analysis and Reporting	Overall risk of bias	Brief justification
Elsamadicy AA 2025	Journal of Clinical Medicine	retrospective registry-based cohort	ACS-NSQIP 2011-2022	Adults >=18 undergoing posterior spinal fusion (PSF) for adult spinal deformity (ASD; n=3,639)	RAI	Low	Low	Moderate	Low	Low	Moderate	Moderate	D3 and D6 are both Moderate; 2 Moderate domains with no High yields overall Moderate; D3 alone (NSQIP-derived adapted RAI-rev) would already trigger Moderate by rule.
Elsamadicy AA 2025	Journal of Neuro-Oncology	retrospective registry-based cohort	ACS-NSQIP 2012-2022	Adults >=18 undergoing laminectomy or corpectomy for extradural metastatic spinal tumors (n=1,796)	mFI + RAI	Low	Low	Moderate	Low	Low	Low	Moderate	One Moderate at D3 (NSQIP-derived tool without explicit validation step in this cohort); all other domains Low; per rule, 1 Moderate at D3 = Moderate overall.
Farid M 2026	Journal of Clinical Neurosci	retrospective registry-based cohort	ACS-NSQIP 2017-2022	Adults >=18 undergoing cranial neurosurgery	mFI + RAI	Low	Low	Moderate	Low	Low	Low	Moderate	One Moderate at D3 (high-weight domain) with all other domains Low

First author, year	Journal	Study design	Data source / database	Surgical population	Frailty tool (s)	Domain 1: Study Participation	Domain 2: Study Attrition	Domain 3: Prognostic Factor Measurement	Domain 4: Outcome Measurement	Domain 5: Study Confounding	Domain 6: Statistical Analysis and Reporting	Overall risk of bias	Brief justification
	ence			(trauma, tumor, vascular, functional) from ACS-NSQIP 2017-2022; n=77,514; excluded if missing albumin, age, sex, height, or weight									triggers Moderate overall per rule.
Gaston B 2026	Journal of Vascular Surgery	retrospective single-center cohort	Single-center retrospective cohort (Massachusetts General Hospital), Jan-Dec 2023	Adults undergoing open or endovascular lower extremity revascularization at MGH 2023; n=182; 74% critical limb ischemia	mFI + RAI	Low	Low	Moderate	Low	Low	Moderate	Moderate	D3=Moderate (high-weight domain) and D6=Moderate gives 2 Moderates with no High; per rule 2-3 Moderates with D3 Moderate = Moderate overall.
Ghanekar SD 2026	Clinical Neurology and Neurosurgery	retrospective registry-based cohort	ACS-NSQIP 2011–2023	Elderly (age >65) patients undergoing craniotomy/crani	RAI	Low	Low	Moderate	Low	Low	Low	Moderate	One domain (D3) is Moderate and all others are Low; a single Moderate at D3 (high-weight

First author, year	Journal	Study design	Data source / database	Surgical population	Frailty tool (s)	Domain 1: Study Participation	Domain 2: Study Attrition	Domain 3: Prognostic Factor Measurement	Domain 4: Outcome Measurement	Domain 5: Study Confounding	Domain 6: Statistical Analysis and Reporting	Overall risk of bias	Brief justification
				ectomy for subdural hematoma evacuation									domain) triggers an overall Moderate rating per the rubric.
Gilan O 2025	BMC Geriatrics	prospective cohort	single-center (Al-Gomhori Teaching Hospital, Sana'a, Yemen)	Older patients (age >=60) undergoing emergency laparotomy, n=89, single-center Yemen, Jan 2023-Dec 2024	CFS	Moderate	Low	Low	Low	Moderate	High	High	Domain 6 rated High (no AUC/c-statistic for 30-day mortality) triggers overall High risk of bias.
Grudzinski A 2022	British Journal of Anaesthesia	retrospective registry-based cohort	ICES Ontario population-based administrative health data (linked databases), 2012-2018	Adults >65 undergoing elective noncardiac surgery (orthopaedic, vascular, oncologic) in Ontario, Canada	RAI	Low	Low	Moderate	Low	Low	High	High	D6 = High because only a multivariable baseline+RAI model AUC is reported and no standalone RAI AUC can be isolated.
Grudzinski AL 2023	Annals of	retrospective	ICES Ontario provincial	Adults ≥65 undergoing	RAI	Low	Low	Moderate	Low	Low	High	High	D6 = High because only a multivariable

First author, year	Journal	Study design	Data source / database	Surgical population	Frailty tool (s)	Domain 1: Study Participation	Domain 2: Study Attrition	Domain 3: Prognostic Factor Measurement	Domain 4: Outcome Measurement	Domain 5: Study Confounding	Domain 6: Statistical Analysis and Reporting	Overall risk of bias	Brief justification
	Surgery	registry-based cohort	administrative health data (linked databases), 2012-2018	emergency general surgery (EGS), Ontario Canada									baseline+RAI AUC is reported and no standalone tool-level AUC can be isolated.
Gupta 2026	European Spine Journal	retrospective registry-based cohort	ACS-NSQIP	Adults undergoing elective single-level ACDF or CDR for cervical degenerative disc disease; spine/neurosurgery; ACS-NSQIP 2012-2022; n=97,408 total (matched n=16,974)	RAI	Low	Low	Moderate	Low	Low	Low	Moderate	D3=Moderate (NSQIP-derived without validation); all other domains Low. One Moderate at D3 (high-weight domain) → Overall Moderate per rubric.
Gupta N 2025	Journal of Orthopaedics	retrospective registry-based cohort	ACS-NSQIP 2015–2019	Elderly obese adults (≥65 yr, BMI≥30) undergoing elective primary	mFI + RAI	Low	Low	Moderate	Low	Low	Low	Moderate	One domain (D3) is Moderate due to registry-derived tool without in-study validation;

First author, year	Journal	Study design	Data source / database	Surgical population	Frailty tool (s)	Domain 1: Study Participation	Domain 2: Study Attrition	Domain 3: Prognostic Factor Measurement	Domain 4: Outcome Measurement	Domain 5: Study Confounding	Domain 6: Statistical Analysis and Reporting	Overall risk of bias	Brief justification
				total joint arthroplasty (THA or TKA) for degenerative joint disease									per the rubric a single Moderate at D3 (high-weight domain) yields an overall Moderate rating.
Gupta N 2025	JAMA Network Open	retrospective cross-sectional registry-based cohort	ACS-NSQIP 2015-2019	Adults >=65 with traumatic hip fracture undergoing surgical fixation, hemiarthroplasty, or THA; ACS-NSQIP 2015-2019; n=114,359	mFI + RAI	Low	Low	Moderate	Low	Low	Low	Moderate	One Moderate at Domain 3 (NSQIP-derived frailty tools without paper-specific validation) with no High domains; one Moderate at D3 = Moderate overall RoB per rubric.
Hall DE 2017	JAMA Surgery	prospective cohort quality improvement study (before-after design)	VASQIP single-center (VA Nebraska-Western Iowa Health Care System) 2007-2014	Adults undergoing major elective noncardiac surgery at a single VA medical center (all 12 surgical	RAI	Moderate	Low	Moderate	Low	Moderate	High	High	D6 = High because no standalone tool-level AUC/C-statistic for 30-day mortality is reported; the study is a QI before-after implementation

First author, year	Journal	Study design	Data source / database	Surgical population	Frailty tool (s)	Domain 1: Study Participation	Domain 2: Study Attrition	Domain 3: Prognostic Factor Measurement	Domain 4: Outcome Measurement	Domain 5: Study Confounding	Domain 6: Statistical Analysis and Reporting	Overall risk of bias	Brief justification
				specialties)									study, not a diagnostic accuracy study.
Hall DE 2019	JAMA Surgery	retrospective cohort	VASQIP April 2010 - March 2014	Veterans undergoing noncardiac surgical procedures at VA hospitals (n=432,828)	RAI	Low	Low	Moderate	Low	Low	Moderate	Moderate	D3 and D6 are both Moderate; 2 Moderate domains with no High yields overall Moderate by rubric rule.
Hall DE 2020	JAMA Surgery	retrospective national cohort	ACS-NSQIP and VASQIP 2010-2014	Adults >=18 undergoing noncardiac surgery across 9 specialties in NSQIP (n=2,339,031) and VASQIP (n=426,578)	RAI	Low	Low	Moderate	Low	Low	Moderate	Moderate	D3 and D6 are both Moderate; 2 Moderate domains with no High yields overall Moderate by rubric rule.
Hall DE 2023	JAMA	retrospective national cohort with coarsened exact	VASQIP and ACS-NSQIP 2016-2019	Female patients >=18 undergoing noncardiac surgery across 9	RAI	Low	Low	Moderate	Low	Low	High	High	D6 is High because no standalone AUC/C-statistic for RAI predicting 30-day mortality is

First author, year	Journal	Study design	Data source / database	Surgical population	Frailty tool (s)	Domain 1: Study Participation	Domain 2: Study Attrition	Domain 3: Prognostic Factor Measurement	Domain 4: Outcome Measurement	Domain 5: Study Confounding	Domain 6: Statistical Analysis and Reporting	Overall risk of bias	Brief justification
		matching		specialties in VASQIP and NSQIP									reported; study is a comparative effectiveness study, not a frailty discrimination study.
Hao Y 2025	BMC Geriatrics	retrospective cohort	single-center (Chinese PLA General Hospital, First Medical Center, China)	Older patients (age >=65) undergoing radical esophagectomy, n=699, single-center China, Jan 2014-Jan 2017	mFI	Low	Moderate	Moderate	Moderate	Low	Moderate	High	Rated high overall because four domains were judged moderate according to the prespecified overall judgment rule.
Hermann CE 2022	Gynecologic Oncology Reports	retrospective registry-based cohort	ACS-NSQIP 2015-2017	Women undergoing hysterectomy for benign or malignant gynecologic indications (n=80,293)	mFI	Low	Low	Moderate	Low	Low	Low	Moderate	D3 is Moderate (NSQIP-derived mFI-5 without in-paper validation); any Moderate at D3 yields overall Moderate by rubric rule.

First author, year	Journal	Study design	Data source / database	Surgical population	Frailty tool (s)	Domain 1: Study Participation	Domain 2: Study Attrition	Domain 3: Prognostic Factor Measurement	Domain 4: Outcome Measurement	Domain 5: Study Confounding	Domain 6: Statistical Analysis and Reporting	Overall risk of bias	Brief justification
Hinojosa-Gonzalez D 2025	Journal of Robotic Surgery	retrospective registry-based cohort	ACS-NSQIP 2019-2022 (nephrectomy-targeted PUFs)	Adults undergoing minimally invasive (laparoscopic or robotic) radical nephrectomy (n=7,324)	mFI	Low	Low	Moderate	Low	Moderate	Moderate	Moderate	Three Moderate ratings (D3, D5, D6) with no High; 2-3 Moderate = Moderate overall.
Ikram MA 2022	The Bone and Joint Journal	prospective observational cohort	single-center (University of Nottingham / Queen's Medical Centre, UK)	Fragility hip fracture patients aged >65 years, n=1255, single-center UK, Apr 2018-Dec 2019	CFS	Moderate	Moderate	Low	Low	Moderate	Low	Moderate	Three domains rated Moderate (D1 participation, D2 attrition, D5 confounding) with no High; 2-3 Moderate without a High triggers overall Moderate per rule.
Isand KG 2023	World Journal of Surgery	retrospective single-centre cohort (NELA database)	NELA database, Oxford University Hospitals NHS Trust,	Adults undergoing emergency laparotomy at Oxford University	mFI	Low	Moderate	Low	Low	Moderate	Moderate	Moderate	Three Moderate ratings (D2, D5, D6) with no High; 2-3 Moderate = Moderate overall.

First author, year	Journal	Study design	Data source / database	Surgical population	Frailty tool (s)	Domain 1: Study Participation	Domain 2: Study Attrition	Domain 3: Prognostic Factor Measurement	Domain 4: Outcome Measurement	Domain 5: Study Confounding	Domain 6: Statistical Analysis and Reporting	Overall risk of bias	Brief justification
			2018-2021 (single-centre)	Hospitals NHS Trust, 2018-2021 (n=793)									
Kazim SF 2022	Neurospine	retrospective registry-based cohort	ACS-NSQIP 2015-2019	Adults ≥18 undergoing resection of extradural, intradural extramedullary, or intramedullary primary or metastatic spinal tumors	mFI	Low	Low	Moderate	Low	Moderate	Low	Moderate	D3 is Moderate (NSQIP-derived mFI-5 without in-paper validation) and D5 is Moderate (ROC analysis unadjusted for tumor type/location); 2 Moderate domains with no High → overall Moderate.
Khandaker R 2025	Journal of Clinical Anesthesia	retrospective single-center hospital registry cohort	Montefiore Medical Center electronic health record (EMR), January 2016–February 2023	Adults undergoing non-ambulatory (inpatient) surgery at a single tertiary academic center (Montefiore, Bronx NY);	mFI	Low	Low	Moderate	Low	Low	Low	Moderate	One domain (D3) is Moderate due to EHR-reconstructed mFI-5 without explicit validation; the single Moderate is at D3 (a high-weight domain), triggering

First author, year	Journal	Study design	Data source / database	Surgical population	Frailty tool (s)	Domain 1: Study Participation	Domain 2: Study Attrition	Domain 3: Prognostic Factor Measurement	Domain 4: Outcome Measurement	Domain 5: Study Confounding	Domain 6: Statistical Analysis and Reporting	Overall risk of bias	Brief justification
				mixed non-cardiac surgical specialties									an overall Moderate per the rubric.
Leigh 2022	Annals of Surgical Oncology	retrospective registry-based cohort	ACS-NSQIP (hepatectomy-targeted)	Adults undergoing elective hepatectomy for colorectal liver metastases (CRLM); general/HPB surgery; NSQIP hepatectomy-targeted database 2014-2019; n=8,477	RAI	Low	Low	Moderate	Low	Low	High	High	D6=High triggers High overall regardless of other domains.
Levy I 2017	Urologic Oncology: Seminars and Original Investiga	retrospective registry-based cohort	ACS-NSQIP 2008–2014	Adults undergoing robot-assisted radical prostatectomy (RARP) for prostate cancer	mFI	Low	Low	Moderate	Low	Low	Moderate	Moderate	Two domains are Moderate (D3 and D6); two Moderate ratings with no High domain yields an overall Moderate per the rubric.

First author, year	Journal	Study design	Data source / database	Surgical population	Frailty tool (s)	Domain 1: Study Participation	Domain 2: Study Attrition	Domain 3: Prognostic Factor Measurement	Domain 4: Outcome Measurement	Domain 5: Study Confounding	Domain 6: Statistical Analysis and Reporting	Overall risk of bias	Brief justification
	tions												
Maegawa FB 2022	HPB	retrospective registry-based cohort	ACS-NSQIP liver-targeted module 2014-2019	Adults undergoing elective hepatectomy for liver tumors; NSQIP liver-targeted module 2014-2019; n=24,150	mFI	Low	Low	Moderate	Low	Low	High	High	D6=High (no standalone mFI AUC for 30-day mortality; only multivariable ALBI+mFI AUC reported) triggers High overall per rule (any High domain = High overall).
Man J 2021	Journal of Surgical Research	retrospective registry-based cohort	ACS-NSQIP 2011-2018	Adults undergoing elective open or endovascular AAA repair (n=4,106 open + 11,733 EVAR)	RAI	Moderate	Low	Moderate	Low	Moderate	Low	Moderate	D3 is Moderate (registry-derived RAI-A with acknowledged approximations for cognitive and living status variables) and D5 is Moderate (univariate RAI-A model without key covariate adjustment); D1

First author, year	Journal	Study design	Data source / database	Surgical population	Frailty tool (s)	Domain 1: Study Participation	Domain 2: Study Attrition	Domain 3: Prognostic Factor Measurement	Domain 4: Outcome Measurement	Domain 5: Study Confounding	Domain 6: Statistical Analysis and Reporting	Overall risk of bias	Brief justification
													also Moderate; 3 Moderate domains with no High → overall Moderate.
Mau E 2025	Bone & Joint Open	retrospective registry-based cohort	ACS-NSQIP 2016-2020	Hip fracture patients aged >=60 undergoing surgical fixation, n=53,959, ACS-NSQIP 2016-2020	mFI + RAI	Low	Low	Moderate	Low	Low	Low	Moderate	One Moderate at Domain 3 (NSQIP-derived frailty tools without paper-specific validation) with no High domains; one Moderate at D3 = Moderate overall RoB per rubric.
McConaghy 2023	Archives of Orthopaedic and Trauma Surgery	retrospective registry-based cohort	ACS-NSQIP	Adults undergoing elective THA (n=202,488) or TKA (n=230,823); orthopedic surgery; ACS-NSQIP 2011-2019	mFI	Low	Low	Moderate	Low	Low	Moderate	Moderate	D3=Moderate (NSQIP-derived without validation) and D6=Moderate. Two Moderate domains, neither triggering High alone. Per rubric: 2–3 Moderate (no High) → Overall

First author, year	Journal	Study design	Data source / database	Surgical population	Frailty tool (s)	Domain 1: Study Participation	Domain 2: Study Attrition	Domain 3: Prognostic Factor Measurement	Domain 4: Outcome Measurement	Domain 5: Study Confounding	Domain 6: Statistical Analysis and Reporting	Overall risk of bias	Brief justification
													Moderate.
McIsaac DI 2021	Anesthesia and Analgesia	retrospective registry-based cohort with Bayesian analysis	ACS-NSQIP 2010-2016	Adults undergoing inpatient small/large bowel surgery 2010-2015 (derivation, n~43,000) and intermediate/high-risk noncardiac surgery 2016 (validation); total n=50,630 reported	mFI + RAI	Low	Low	Moderate	Low	Low	High	High	Domain 6 rated High (no AUC/c-statistic for 30-day mortality) triggers overall High risk of bias.
McQueston C 2026	Journal of Surgical Research	retrospective single-center cohort	VASQIP single-center (VA Medical Center Buffalo, NY)	Mixed noncardiac surgical patients admitted to SICU at VA Medical Center, Jan 2019-Dec 2022; n=541	RAI	Low	Low	Moderate	Low	Low	Low	Moderate	One Moderate at D3 (high-weight domain) with all other domains Low triggers Moderate overall per rule.

First author, year	Journal	Study design	Data source / database	Surgical population	Frailty tool (s)	Domain 1: Study Participation	Domain 2: Study Attrition	Domain 3: Prognostic Factor Measurement	Domain 4: Outcome Measurement	Domain 5: Study Confounding	Domain 6: Statistical Analysis and Reporting	Overall risk of bias	Brief justification
Modi M 2026	Indian Journal of Orthopaedics	retrospective registry-based cohort	ACS-NSQIP (data years not specified in paper)	Patients undergoing surgical fixation of distal femur fractures (CPT 27511, 27513, 27514), n=2638, ACS-NSQIP	mFI + RAI	Low	Low	Moderate	Low	Moderate	Low	Moderate	Two domains rated Moderate (D3 tool measurement, D5 confounding) with D3 being Moderate; 1 Moderate at D3 alone triggers at least Moderate, confirmed by 2 Moderate total per rule.
Moya A 2023	Plastic and Reconstructive Surgery Global Open	retrospective registry-based cohort	ACS-NSQIP 2012-2020	Adults >=18 undergoing cranioplasty surgery; ACS-NSQIP 2012-2020; n=2864	mFI + RAI	Low	Low	Moderate	Low	Low	Low	Moderate	One Moderate at Domain 3 (NSQIP-derived administrative frailty scoring without paper-specific validation) with no High domains; one Moderate at D3 = Moderate overall RoB per rubric.
Musharba	European	retrospectiv	ACS-NSQIP	Adults ≥18	mFI	Low	Low	Moder	Low	Mode	Modera	Mod	Three domains

First author, year	Journal	Study design	Data source / database	Surgical population	Frailty tool (s)	Domain 1: Study Participation	Domain 2: Study Attrition	Domain 3: Prognostic Factor Measurement	Domain 4: Outcome Measurement	Domain 5: Study Confounding	Domain 6: Statistical Analysis and Reporting	Overall risk of bias	Brief justification
sh 2023	Spine Journal	e registry-based cohort		undergoing surgery for metastatic spine disease; neurosurgery/spine oncology; NSQIP 2010-2018; n=1,195				ate		rate	te	erate	rated Moderate (D3, D5, D6). Per rubric: 2–3 Moderate (no High) → Overall Moderate.
Narula S 2020	Bone & Joint Open	retrospective cohort	single-center (Fiona Stanley Hospital, Perth, Western Australia)	Proximal femur fracture patients undergoing surgical repair, n=509, single-center Australia	CFS	Low	Low	Moderate	Low	Moderate	High	High	Domain 6 is High (no 30-day AUC reported, only one-year AUC); any High domain triggers High overall RoB per rubric.
Not reported (Kaiser Permanente/KPNC study) 2022	JAMA Surgery	retrospective external validation cohort	Kaiser Permanente Northern California EHR with NSQIP sampling,	Adults >50 undergoing abdominal surgery at 20 KPNC medical centers sampled for NSQIP	mFI + RAI	Low	Low	Moderate	Low	Low	Low	Moderate	D3 is Moderate (RAI adapted from EHR using code replacements without in-paper validation); any Moderate at D3

First author, year	Journal	Study design	Data source / database	Surgical population	Frailty tool (s)	Domain 1: Study Participation	Domain 2: Study Attrition	Domain 3: Prognostic Factor Measurement	Domain 4: Outcome Measurement	Domain 5: Study Confounding	Domain 6: Statistical Analysis and Reporting	Overall risk of bias	Brief justification
			2010-2020	reporting (n=37,186 procedures)									yields overall Moderate by rubric rule.
Olgun Keles G 2026	BMC Surgery	retrospective cohort	single-center (Giresun Training and Research Hospital, Turkey)	Nonagenarian patients (age >=90) undergoing mixed non-cardiac surgery (orthopedic 52%, general 21%, urology 19%), n=177, single-center Turkey, Nov 2021-Apr 2023	mFI	Moderate	Low	Low	Low	Moderate	Moderate	Moderate	Three domains rated Moderate (D1 participation, D5 confounding, D6 statistics) with no High; 2-3 Moderate without a High triggers overall Moderate per rule.
Owodunni 2023	The Spine Journal	retrospective registry-based cohort	ACS-NSQIP	Elderly (>=65 years), obese (BMI>=30) adults undergoing spine surgery from ACS-NSQIP	mFI + RAI	Low	Low	Moderate	Low	Low	Low	Moderate	D3=Moderate (NSQIP-derived without validation); all other domains Low. One Moderate at D3 (high-weight domain) → Overall

First author, year	Journal	Study design	Data source / database	Surgical population	Frailty tool (s)	Domain 1: Study Participation	Domain 2: Study Attrition	Domain 3: Prognostic Factor Measurement	Domain 4: Outcome Measurement	Domain 5: Study Confounding	Domain 6: Statistical Analysis and Reporting	Overall risk of bias	Brief justification
				2012-2020									Moderate per rubric.
Paiz CC 2024	World Neurosurgery: X	retrospective registry-based cohort	ACS-NSQIP 2012-2020	Adults undergoing cranial or spine neurosurgery with subsequent major Clavien-Dindo III-IV complications (n=19,096)	mFI + RAI	Moderate	Low	Moderate	Low	Moderate	Low	Moderate	D1, D3, and D5 are all Moderate (3 Moderate domains); 3 Moderate with no High yields overall Moderate by rubric rule.
Palaniappan M 2022	Colorectal Disease	prospective cohort	ELLSA (Emergency Laparoscopic and Laparotomy Scottish Audit), 18 Scottish acute surgical units	Adults >=18 undergoing emergency laparotomy/laparoscopy, n=2246, 18 Scottish units, Nov 2017-Oct 2018	CFS	Low	Moderate	Moderate	Low	Low	Low	Moderate	Two Moderate ratings (D2: high CFS missingness; D3: 7-point CFS version and high missing data) with no High domains; 2-3 Moderate = Moderate overall RoB per rubric.
Pean C 2024	The Journal	retrospective	ACS-NSQIP 2013-2020	Adults >=18 undergoing	mFI	Low	Low	Moderate	Low	Low	Moderate	Moderate	D3=Moderate (high-weight) and

First author, year	Journal	Study design	Data source / database	Surgical population	Frailty tool (s)	Domain 1: Study Participation	Domain 2: Study Attrition	Domain 3: Prognostic Factor Measurement	Domain 4: Outcome Measurement	Domain 5: Study Confounding	Domain 6: Statistical Analysis and Reporting	Overall risk of bias	Brief justification
	of Arthroplasty	registry-based cohort		revision THA or TKA; ACS-NSQIP 2013-2020; n=22,836; randomly split 80:20 training/validation cohorts									D6=Moderate gives 2 Moderates with no High; per rule 2-3 Moderates with D3 Moderate = Moderate overall.
Prvulovic 2025	European Spine Journal	retrospective registry-based cohort	ACS-NSQIP	Adults ≥18 undergoing surgical fixation of thoracolumbar vertebral fractures; neurosurgery/spine; NSQIP 2015-2019; n=42,230	mFI + RAI	Low	Low	Moderate	Low	Low	Low	Moderate	D3=Moderate (NSQIP-derived without validation); all other domains Low. One Moderate at D3 (high-weight domain) → Overall Moderate per rubric.
Prvulovic ST 2025	Global Spine Journal	retrospective registry-based cohort	ACS-NSQIP 2015–2020	Adults (≥18 yr) undergoing spine surgery (broad spectrum, CPT codes	mFI + RAI	Low	Low	Moderate	Low	Low	Low	Moderate	One domain (D3) is Moderate due to registry-derived tool without in-study validation;

First author, year	Journal	Study design	Data source / database	Surgical population	Frailty tool (s)	Domain 1: Study Participation	Domain 2: Study Attrition	Domain 3: Prognostic Factor Measurement	Domain 4: Outcome Measurement	Domain 5: Study Confounding	Domain 6: Statistical Analysis and Reporting	Overall risk of bias	Brief justification
				22010–22855 and 62380–63707)									single Moderate at D3 (high-weight domain) triggers Moderate overall per the rubric.
Rothenberg KA 2020	Annals of Vascular Surgery	retrospective registry-based cohort	ACS-NSQIP 2007-2013	Adults undergoing major elective vascular surgery (6 categories: carotid revascularization, AAA repair, suprainguinal/infrainguinal revascularization, TAA, TAAA repair); emergency cases excluded	RAI	Low	Low	Moderate	Low	Moderate	Moderate	Moderate	D3 is Moderate (NSQIP-derived RAI with acknowledged missing cognitive data) and D5 and D6 are also Moderate; 3 Moderate domains with no High → overall Moderate.
Sabet A 2026	Arthroplasty	retrospective registry-based cohort	ACS-NSQIP 2015-2021	Female patients aged 65-89 undergoing inpatient total	mFI + RAI	Moderate	Low	Moderate	Low	Moderate	Low	Moderate	Three domains rated Moderate (D1 participation, D3 tool measurement,

First author, year	Journal	Study design	Data source / database	Surgical population	Frailty tool (s)	Domain 1: Study Participation	Domain 2: Study Attrition	Domain 3: Prognostic Factor Measurement	Domain 4: Outcome Measurement	Domain 5: Study Confounding	Domain 6: Statistical Analysis and Reporting	Overall risk of bias	Brief justification
				shoulder arthroplasty (TSA, CPT 23472), n=11,965, ACS-NSQIP 2015-2021									D5 confounding) with no High; 2-3 Moderate including D3 triggers overall Moderate per rule.
Sabet CJ 2025	The Knee	retrospective registry-based cohort	ACS-NSQIP 2015-2021	Adults undergoing elective unicompartmental knee arthroplasty (UKA); NSQIP 2015-2021; n=9358 (1709 excluded for missing outcome data from 11,067 initially identified)	mFI + RAI	Moderate	Moderate	Moderate	Moderate	Low	Moderate	High	D1=Moderate, D2=Moderate, D3=Moderate (high-weight), D4=Moderate, D6=Moderate gives 5 Moderates; per rule >=4 Moderates = High.
Sabet CJ 2026	The Surgeon	retrospective registry-bas	ACS-NSQIP 2015–2021	Adult male patients undergoing	mFI + RAI	Low	Low	Moderate	Low	Low	High	High	Domain 6 is High (no standalone frailty-tool AUC

First author, year	Journal	Study design	Data source / database	Surgical population	Frailty tool (s)	Domain 1: Study Participation	Domain 2: Study Attrition	Domain 3: Prognostic Factor Measurement	Domain 4: Outcome Measurement	Domain 5: Study Confounding	Domain 6: Statistical Analysis and Reporting	Overall risk of bias	Brief justification
		ed cohort		surgical treatment for isolated ankle fractures									for 30-day mortality reported), which automatically yields an overall High RoB.
Sbaih O 2025	Global Spine Journal	retrospective registry-based cohort	ACS-NSQIP 2015–2020	Adults (≥18–90 yr) undergoing occipitocervical fusion (OCF), both elective and non-elective	mFI + RAI	Low	Low	Moderate	Low	Low	Low	Moderate	One domain (D3) is Moderate due to registry-derived tool without in-study validation; single Moderate at D3 (high-weight domain) triggers Moderate overall per the rubric.
Skandalakis GP 2023	Neurosurgical Focus	retrospective registry-based cohort	ACS-NSQIP 2012-2020	Adults undergoing surgical resection of intracranial brain metastases (cranial neurosurgery)	mFI + RAI	Moderate	Low	Moderate	Low	Moderate	Low	Moderate	D3 is Moderate (NSQIP registry-derived frailty without in-paper validation) and D5 is Moderate (ROC analysis unadjusted for

First author, year	Journal	Study design	Data source / database	Surgical population	Frailty tool (s)	Domain 1: Study Participation	Domain 2: Study Attrition	Domain 3: Prognostic Factor Measurement	Domain 4: Outcome Measurement	Domain 5: Study Confounding	Domain 6: Statistical Analysis and Reporting	Overall risk of bias	Brief justification
													case-mix; tumor type not captured); 2 Moderate domains with no High → overall Moderate.
Snitkjaer C 2024	BJS Open	prospective single-centre cohort	Copenhagen University Hospital Herlev, Denmark (single-centre, Oct 2020-Aug 2022)	Adults (>=18) undergoing major emergency abdominal surgery (MEAS) at Copenhagen University Hospital Herlev (n=520)	mFI	Low	Low	Low	Moderate	Low	High	High	Domain 6 rated High (no AUC/c-statistic for CFS predicting 30-day mortality); any High = High overall.
Soni C 2025	Journal of the AAOS Global Research and Reviews	retrospective registry-based cohort	ACS-NSQIP 2015-2020	Adults >=18 undergoing surgical treatment for orthopaedic fractures (forearm, humerus, pelvis,	mFI + RAI	Low	Low	Moderate	Low	Low	Low	Moderate	D3 is Moderate (NSQIP-derived RAI-Rev without in-paper validation); any Moderate at D3 yields overall Moderate by rubric

First author, year	Journal	Study design	Data source / database	Surgical population	Frailty tool (s)	Domain 1: Study Participation	Domain 2: Study Attrition	Domain 3: Prognostic Factor Measurement	Domain 4: Outcome Measurement	Domain 5: Study Confounding	Domain 6: Statistical Analysis and Reporting	Overall risk of bias	Brief justification
				acetabulum, femur, tibia, hindfoot)									rule.
Spitzer H 2022	Journal of Surgical Research	retrospective registry-based cohort	ACS-NSQIP pancreas-targeted Participant Use File 2014-2017	Adults undergoing distal pancreatectomy or pancreatoduodenectomy (n=22,123)	mFI	Moderate	Low	Moderate	Low	Moderate	Low	Moderate	D3 is Moderate (NSQIP-derived mFI-5 without in-paper validation) and D5 is Moderate (univariate mFI-5 model without key confounders adjusted); D1 also Moderate; 3 Moderate domains with no High → overall Moderate.
Sánchez Arteaga 2021	European Journal of Trauma and Emergency Surgery	prospective cohort	single-center	Patients ≥70 years undergoing emergency abdominal surgery; single-center Spain (Hospital Universitario	CFS	Moderate	Low	Moderate	Low	Moderate	Moderate	High	Four domains rated Moderate (D1, D3, D5, D6). Per rubric: ≥4 Moderate → Overall High.

First author, year	Journal	Study design	Data source / database	Surgical population	Frailty tool (s)	Domain 1: Study Participation	Domain 2: Study Attrition	Domain 3: Prognostic Factor Measurement	Domain 4: Outcome Measurement	Domain 5: Study Confounding	Domain 6: Statistical Analysis and Reporting	Overall risk of bias	Brief justification
				Virgen del Rocio); September 2017-April 2019; n=92									
Talukder R 2025	Clinical Neurology and Neurosurgery	retrospective registry-based cohort	ACS-NSQIP 2007–2021	Adults undergoing peripheral nerve surgery (neurorrhaphy with nerve grafts)	mFI	Low	Low	Moderate	Low	Low	High	High	Domain 6 is High (no usable AUC for the primary effect of interest), which automatically raises overall RoB to High per the rubric.
Thommen R 2022	World Neurosurgery	retrospective registry-based cohort	ACS-NSQIP 2015–2019	Adults (≥18 yr) undergoing pituitary adenoma resection (transsphenoidal or craniotomy)	mFI	Low	Low	Moderate	Low	Low	High	High	Domain 6 is High (no usable standalone AUC for 30-day mortality), yielding an automatic overall High RoB.
Torres Lima DF 2019	Saudi Journal of Anesthesia	prospective single-centre observational cohort	Centro Hospitalar Sao Joao, Portugal (single-centre	Elderly (>=60 years) patients undergoing elective non-cardiac	mFI	Moderate	Low	Low	Moderate	Moderate	Low	Moderate	Three Moderate ratings (D1, D4, D5) with no High; 2-3 Moderate = Moderate overall.

First author, year	Journal	Study design	Data source / database	Surgical population	Frailty tool (s)	Domain 1: Study Participation	Domain 2: Study Attrition	Domain 3: Prognostic Factor Measurement	Domain 4: Outcome Measurement	Domain 5: Study Confounding	Domain 6: Statistical Analysis and Reporting	Overall risk of bias	Brief justification
			, May-July 2017)	surgery (general, urology, gynecology, plastic, orthopedic, maxillofacial) at a university hospital in Portugal (n=229)									
Warrier A 2026	The Laryngoscope	retrospective registry-based cohort	ACS-NSQIP 2005-2020	Adults undergoing otologic or neurotologic surgery from ACS-NSQIP 2005-2020 (n=2,862)	mFI + RAI	Low	Low	Moderate	Low	Low	Low	Moderate	One Moderate at D3 (NSQIP-derived RAI-A administrative version without explicit validation in this otologic cohort); all other domains Low; 1 Moderate at D3 = Moderate overall.
Williamson TK 2026	JSES Reviews, Reports, and	retrospective registry-based cohort	ACS-NSQIP 2015-2020	Adults >=60 undergoing ORIF or reverse total shoulder	mFI + RAI	Low	Low	Moderate	Low	Moderate	Low	Moderate	D3 and D5 are both Moderate; 2 Moderate domains with no High yields

First author, year	Journal	Study design	Data source / database	Surgical population	Frailty tool (s)	Domain 1: Study Participation	Domain 2: Study Attrition	Domain 3: Prognostic Factor Measurement	Domain 4: Outcome Measurement	Domain 5: Study Confounding	Domain 6: Statistical Analysis and Reporting	Overall risk of bias	Brief justification
	Techniques			arthroplasty (rTSA) for proximal humerus fractures (n=6,020)									overall Moderate by rubric rule.
Wu J 2021	International Urology and Nephrology	prospective observational cohort (secondary analysis of larger prospective cohort)	single-center (Lancashire Teaching Hospitals NHS Foundation Trust, UK)	Hip fracture patients with CKD G3b-5D, n=397, single-center UK	CFS	Low	Low	Low	Low	Low	Low	Low	All six domains rated Low.
Yang 2024	HPB	retrospective cohort	multi-center (16 hepatobiliary centers, China)	Octogenarians (>=80 years) undergoing hepatectomy for HCC at 16 hepatobiliary centers in China, 2010-2022	CFS	Low	Moderate	Low	Low	Low	High	High	D6=High triggers High overall regardless of other domains.
Yocky AG 2023	Neurosurgery	retrospective	ACS-NSQIP 2012-2020	Octogenarians (≥80 years)	mFI + RAI	Moderate	Low	Moderate	Low	Moderate	Low	Moderate	D3 is Moderate (NSQIP-derived

First author, year	Journal	Study design	Data source / database	Surgical population	Frailty tool (s)	Domain 1: Study Participation	Domain 2: Study Attrition	Domain 3: Prognostic Factor Measurement	Domain 4: Outcome Measurement	Domain 5: Study Confounding	Domain 6: Statistical Analysis and Reporting	Overall risk of bias	Brief justification
	Practice	registry-based cohort		undergoing neurosurgery (spine 76.9%, cranial 20.5%, other 2.7%)									tools without explicit validation) and D5 is Moderate (ROC analysis not adjusted for key case-mix factors); 2 Moderate domains with no High → overall Moderate.
Zaib J 2023	Cureus	retrospective cohort	single-center (Hull Royal Infirmary, UK)	Hip fracture patients aged >=60 at Hull Royal Infirmary UK, January-December 2018, n=628	CFS	Low	Moderate	Moderate	High	Moderate	High	High	Two High domains (D4 and D6: outcome window ambiguous for the AUC; AUC not confirmed as 30-day-specific); any High domain triggers High overall RoB.
Zamanzadeh R 2024	The Journal of Arthroplasty	retrospective registry-based cohort	ACS-NSQIP 2015-2020	Adults undergoing aseptic revision total hip (rTHA) or total knee	mFI	Low	Low	Moderate	Low	Low	Moderate	Moderate	D3=Moderate (high-weight) and D6=Moderate gives 2 Moderates with no High; per rule

First author, year	Journal	Study design	Data source / database	Surgical population	Frailty tool (s)	Domain 1: Study Participation	Domain 2: Study Attrition	Domain 3: Prognostic Factor Measurement	Domain 4: Outcome Measurement	Domain 5: Study Confounding	Domain 6: Statistical Analysis and Reporting	Overall risk of bias	Brief justification
				arthroplasty (rTKA); NSQIP 2015-2020; n=32,069; infection-related revisions excluded									2-3 Moderates with D3 Moderate = Moderate overall.

Risk of bias assessment of the 77 included studies using the Quality In Prognosis Studies (QUIPS) tool. Each row represents one included study. Ratings are presented for the six QUIPS domains (Domain 1, Study Participation; Domain 2, Study Attrition; Domain 3, Prognostic Factor Measurement; Domain 4, Outcome Measurement; Domain 5, Study Confounding; Domain 6, Statistical Analysis and Reporting), with the overall risk-of-bias judgment assigned according to the prespecified decision rules described in the Methods, in which Domains 3 and 4 were weighted as core domains. Domain-level and overall ratings were performed independently by two reviewers (X.Y. and S.D.); discrepancies were resolved through discussion and consensus. The brief justification column summarizes the rationale for the overall rating. The frailty tool column indicates the screening tool(s) assessed in each study; studies that reported both the Modified Frailty Index and the Risk Analysis Index are classified as "mFI + RAI." Abbreviations: ACS-NSQIP, American College of Surgeons National Surgical Quality Improvement Program; AUC, area under the receiver operating characteristic curve; CFS, Clinical Frailty Scale; mFI, Modified Frailty Index; QUIPS, Quality In Prognosis Studies; RAI, Risk Analysis Index; RoB, risk of bias; VASQIP, Veterans Affairs Surgical Quality Improvement Program.

Supplementary Appendix 3. Database Overlap Identification and Overlap-Adjusted Analysis Decision Log

Study: *Comparative Prognostic Discrimination of the Modified Frailty Index and Risk Analysis Index for 30-Day Mortality After Noncardiac Surgery: A Systematic Review and Meta-analysis.*

PROSPERO registration: CRD420261378778. Initial registration: 25 April 2026. Amendment: 12 May 2026.

1. Overview

Several included studies derived their data from the same public surgical registries, raising the possibility of patient-level overlap across multiple publications. This appendix documents the identification of registry-derived studies, the assessment of overlap between studies within each registry, and the decisions made for the overlap-adjusted sensitivity analysis described in the Methods. Overlap exclusion was used only to reduce potential patient-level duplication in meta-analysis and was not interpreted as a judgment of study quality.

2. Overlap decision rule

Two studies were judged to potentially share patient-level data when all of the following held:

- Same registry or database.
- Reported year coverage overlap of ≥50%.
- Surgical specialty / population overlap of ≥50%.

If overlap was judged present, one representative study was retained per overlap group for the overlap-adjusted sensitivity analysis. Retention priority was applied in the following order:

1. Head-to-head mFI vs RAI study (within-cohort comparison preserved).
2. Largest analytic sample size.
3. Most complete reporting of AUC and variance information (95% confidence interval or standard error).
4. Most comprehensive methodological reporting.
5. Most recent publication, if otherwise similar.

All studies, including those excluded from the overlap-adjusted set, remain reported in the main descriptive synthesis and in the QUIPS risk-of-bias table.

Section A. Distribution of included studies by data source

Table S3.1. Distribution of included studies by data source.

Data source / registry	Number of studies	Studies (first author, year)	Notes
ACS-NSQIP	48	Allen M 2023; Bowers C 2023; Bowers C 2023; Branstetter RM IV 2024; Cartagena-Reyes M 2024; Conlon M 2022; Deol E 2024; Ehlert B 2016; Elsamadicy A 2025; Elsamadicy AA 2025; Elsamadicy AA 2025; Farid M 2026; Ghanekar SD 2026; Gupta 2026; Gupta N 2025; Gupta N 2025; Hermann CE 2022; Hinojosa-Gonzalez D 2025; Kazim SF 2022; Leigh 2022; Levy I 2017; Maegawa FB 2022; Man J 2021; Mau E 2025; McConaghy 2023; McIsaac DI 2021; Modi M 2026; Moya A 2023; Musharbash 2023; Owodunni 2023; Paiz CC 2024; Pean C 2024; Prvulovic 2025; Prvulovic ST 2025; Rothenberg KA 2020; Sabet A 2026; Sabet CJ 2025; Sabet CJ 2026; Sbaih O 2025; Skandalakis GP 2023; Soni C 2025; Spitzer H 2022; Talukder R 2025; Thommen R 2022; Warrier A 2026; Williamson TK 2026; Yocky AG 2023; Zamanzadeh R 2024	American College of Surgeons National Surgical Quality Improvement Program; the dominant data source in this review.
VASQIP	3	Hall DE 2017; Hall DE 2019; McQuestion C 2026	Veterans Affairs Surgical Quality Improvement Program.
VASQIP + ACS-NSQIP (combined)	3	Arya 2020; Hall DE 2020; Hall DE 2023	Studies that explicitly draw on both VASQIP and ACS-NSQIP cohorts (e.g., the RAI development paper).
Other multicentre registry (NRD)	1	Courville E 2024	Healthcare Cost and Utilization Project Nationwide Readmissions Database.
Integrated health system cohort / KPNC	1	Not reported (Kaiser Permanente/KPNC study) 2022	Integrated health system cohort; not part of ACS-NSQIP.
Other multicentre registry (ICES)	2	Grudzinski A 2022; Grudzinski AL 2023	Ontario population-based linked administrative

Data source / registry	Number of studies	Studies (first author, year)	Notes
			health data.
Multicentre non-registry	4	Diao Y 2025; Isand KG 2023; Palaniappan M 2022; Yang 2024	Author-assembled multi-centre cohorts not derived from a national registry.
Single-centre prospective	8	Aakre E 2024; Darvall J 2020; Gilan O 2025; Ikram MA 2022; Snitkjaer C 2024; Sánchez Arteaga 2021; Torres Lima DF 2019; Wu J 2021	Single-institution prospective cohorts.
Single-centre retrospective	7	Chen X 2024; Gaston B 2026; Hao Y 2025; Khandaker R 2025; Narula S 2020; Olgun Keles G 2026; Zaib J 2023	Single-institution retrospective cohorts.
Total	77	—	—

Section B. Overlap groups and retention decisions

Table S3.2. Overlap groups and retention decisions for the overlap-adjusted sensitivity analysis.

Overlap group ID	Registry / database	Overlapping studies	Reported year ranges	Surgical population overlap	Retained study for overlap-adjusted analysis	Excluded studies for overlap-adjusted analysis	Retention rationale	Tool family affected
OG-1	ACS-NSQIP	Prvulovic ST 2025 (broad spine); Bowers C 2023 (all-spine cohort); Bowers C 2023 (ACDF subset); Sbaih O 2025	2015–2019/2020	Adult spine surgery (broad spine cohort with subprocedure subsets — ACDF, occipitocervical fusion)	Prvulovic ST 2025 (broad spine)	Bowers C 2023 (all-spine cohort); Bowers C 2023 (ACDF subset); Sbaih O 2025	Largest analytic sample (n=360,133), head-to-head mFI vs RAI reported, AUC with 95% CI for both tools; Bowers all-spine and ACDF subset and Sbaih OCF subset are nested within or share the same NSQIP source years.	mFI + RAI
OG-2	ACS-NSQIP	Conlon M 2022; Prvulovic S 2025 (thoracolumbar fractures)	2015–2019	Adult spine trauma (traumatic spinal injury / thoracolumbar vertebral fractures)	Conlon M 2022	Prvulovic S 2025 (thoracolumbar fractures)	Head-to-head three-tool comparison (RAI-A, RAI-Rev, mFI-5) with AUC and 95% CI for each, taking precedence over the larger but tool-restricted thoracolumbar-fracture cohort.	mFI + RAI
OG-3	ACS-NSQIP	Gupta N 2025 (hip fracture); Mau E 2025; Soni C 2025	2015–2020	Adult hip-fracture / orthopaedic trauma (hip fracture and orthopaedic	Gupta N 2025 (hip fracture)	Mau E 2025; Soni C 2025	Head-to-head mFI vs RAI in a hip-fracture-specific cohort with	mFI + RAI

Overlap group ID	Registry / database	Overlapping studies	Reported year ranges	Surgical population overlap	Retained study for overlap-adjusted analysis	Excluded studies for overlap-adjusted analysis	Retention rationale	Tool family affected
				trauma cohorts in which femur fractures predominate)			the largest hip-fracture-specific analytic sample (n=114,359), AUC with 95% CI for both tools; Mau hip-fracture cohort and the broader Soni orthopaedic trauma cohort overlap the same NSQIP years and patient pool.	
OG-4	ACS-NSQIP	Rothenberg KA 2020; Ehlert B 2016	2006–2013	Adult major elective vascular surgery (carotid, open/endovascular AAA, lower-extremity revascularisation)	Rothenberg KA 2020	Ehlert B 2016	Largest analytic sample (n=139,569) with reported standard error for the AUC; Ehlert mFI-11 vascular cohort overlaps nearly the same NSQIP vascular years and the same procedure mix.	mFI / RAI (single-tool studies in this group)

Although OG-1 and OG-2 both originated from ACS-NSQIP spine-related cohorts and shared overlapping calendar years, they were treated as separate overlap groups because OG-1

represented broad spine surgery and procedure-specific subsets, whereas OG-2 represented trauma-specific spine cohorts. Under the prespecified rule, they were not combined because the surgical population overlap was not judged to meet the ≥50% threshold at the subgroup level.

Section C. Comparison of primary and overlap-adjusted analysis sets

Among the 77 studies included in the systematic review, 44 mFI/RAI AUC observations from 26 unique studies contributed to the primary pooled AUC analysis. The overlap-adjusted analysis was applied to this primary AUC-ready subset, not to the full systematic-review pool.

Table S3.3. Comparison of primary and overlap-adjusted analysis sets.

Counts in this table refer to the primary AUC-ready mFI/RAI analysis dataset, rather than to all studies included in the systematic review.

Tool family	Primary analysis rows	Primary unique studies	Overlap-adjusted rows	Overlap-adjusted unique studies	Studies excluded from overlap-adjusted analysis	Notes
mFI	22	19	15	14	Sbaih O 2025; Mau E 2025; Ehlert B 2016; Bowers C 2023 (all-spine cohort); Bowers C 2023 (ACDF subset)	Includes mFI-5, mFI-11, and unspecified-version mFI rows.
RAI	22	19	16	13	Sbaih O 2025; Mau E 2025; Bowers C 2023 (all-spine cohort); Bowers C 2023 (ACDF subset); Soni C 2025; Prvulovic S 2025 (thoracolumbar fractures)	Includes RAI, RAI-Rev, RAI-A, and RAI-C rows.
mFI + RAI head-to-head (same cohort)	24	12	16	8	Sbaih O 2025; Mau E 2025; Bowers C 2023 (all-spine cohort); Bowers C 2023 (ACDF subset)	Each head-to-head study contributed one mFI row and one RAI row. Within-cohort head-to-head comparison; descriptive only (no pooled paired-difference SE because

Tool family	Primary analysis rows	Primary unique studies	Overlap-adjusted rows	Overlap-adjusted unique studies	Studies excluded from overlap-adjusted analysis	Notes
						within-study covariance is not reported in source studies).
Overall (mFI + RAI primary pool)	44	26	31	19	Sbaih O 2025; Mau E 2025; Ehlert B 2016; Bowers C 2023 (all-spine cohort); Bowers C 2023 (ACDF subset); Soni C 2025; Prvulovic S 2025 (thoracolumbar fractures)	7 studies excluded for overlap; reasons in Table S3.2.

Brief interpretation

The systematic review included 77 studies overall. Among these, 44 mFI/RAI AUC observations from 26 unique studies contributed to the primary pooled AUC analysis. The overlap-adjusted sensitivity analysis was applied to this primary AUC-ready dataset and retained 31 AUC observations from 19 unique studies after excluding 7 studies with potential overlap. These exclusions reflect potential patient-level duplication within ACS-NSQIP source data and should not be interpreted as a judgment of methodological inferiority. Because studies reporting both mFI and RAI contributed one study-tool row per tool, exclusion of 7 overlapping studies resulted in removal of 13 AUC rows from the primary AUC-ready dataset. Pooled estimates are not reported in this appendix; the primary and overlap-adjusted pooled AUC results are reported in the main Results and in Supplementary Appendix 4.

End of Supplementary Appendix 3.

Supplementary Appendix 4. Supplementary and Sensitivity Analyses

Study: *Comparative Prognostic Discrimination of the Modified Frailty Index and Risk Analysis Index for 30-Day Mortality After Noncardiac Surgery: A Systematic Review and Meta-analysis.*

PROSPERO registration: CRD420261378778. Initial registration: 25 April 2026. Amendment: 12 May 2026.

Methods overview

Discrimination of each frailty tool for 30-day all-cause mortality was summarised on the logit-AUC scale. For studies that reported AUC point estimates without a 95% confidence interval or standard error but with both sample size and the number of 30-day deaths, the standard error was imputed using the Hanley–McNeil (1982) method and converted to the logit-AUC scale by the delta method. Random-effects pooling used the REML estimator for between-study variance (τ^2); confidence intervals around the pooled estimate were computed with the Hartung–Knapp adjustment for $k \geq 2$, with t-distribution quantiles on $k - 1$ degrees of freedom. Heterogeneity was quantified by I^2. Prediction intervals were calculated, where $k \geq 3$, on t with $k - 2$ degrees of freedom. All pooled point estimates and intervals were back-transformed to the AUC scale for reporting. No additional study was added or removed at this stage; sensitivity analyses operated on the primary AUC-ready dataset and the overlap-adjusted subset defined in Supplementary Appendix 3.

Section A. Primary AUC-ready study-tool entries

Table S4.1. Study-tool entries included in the primary pooled AUC analysis.

All entries are mFI- or RAI-derived AUC observations from the primary AUC-ready dataset (44 study-tool rows; 26 unique studies). Variance source codes: 'reported_CI' = AUC and 95% CI taken as reported; 'reported_SE' = AUC SE taken as reported and converted via delta method; 'estimated_SE' = AUC SE imputed via Hanley–McNeil from sample size and 30-day death count and converted via delta method.

A dash indicates that the number of 30-day deaths was not reported or was not required for SE derivation because variance information was directly reported.

First author, year	Tool family	Tool subtype	Data source	Sample size	30-day deaths	AUC (95% CI)	Variance source
Arya S 2020	RAI	RAI-A	VASQIP; ACS-NSQIP; single-center VA	480731	—	0.842 (0.835–0.848)	reported_CI
Arya S 2020	RAI	RAI-rev	VASQIP; ACS-NSQIP; single-center VA	480731	—	0.864 (0.858–0.869)	reported_CI
Arya S 2020	RAI	RAI-rev	VASQIP; ACS-NSQIP; single-center VA	480731	—	0.870 (0.867–0.873)	reported_CI
Author not specified (Kaiser Permanente electronic frailty metrics study) 2022	RAI	RAI	KPNC (Kaiser Permanente Northern Ca	37186	—	0.690 (0.670–0.710)	reported_CI
Bowers C 2023	RAI	RAI	ACS-NSQIP	292225	—	0.802 (0.800–0.803)	reported_CI
Bowers C 2023	RAI	RAI	ACS-NSQIP	61441	—	0.855 (0.852–0.858)	reported_CI
Branstetter RM IV 2024	RAI	RAI	NSQIP	315725	—	0.790 (0.782–0.800)	reported_CI
Conlon M 2022	RAI	RAI-A	ACS-NSQIP	6571	—	0.768 (0.758–0.779)	reported_CI
Conlon M 2022	RAI	RAI-rev	ACS-NSQIP	6571	—	0.819 (0.810–0.829)	reported_CI
Courville E 2024	RAI	RAI	Nationwide Readmissions Database (N	9319	—	0.690 (0.680–0.700)	reported_CI
Gupta N 2025	RAI	RAI	NSQIP	169065	—	0.700 (0.696–0.700)	reported_CI
Gupta N 2025	RAI	RAI	ACS-NSQIP	114359	—	0.730 (0.720–0.730)	reported_CI
Gupta N 2026	RAI	RAI	ACS-NSQIP	97408	—	0.850 (0.820–0.890)	reported_CI
Mau E 2025	RAI	RAI-rev	ACS-NSQIP	53959	—	0.720 (0.710–0.730)	reported_CI
Modi M 2026	RAI	RAI	ACS-NSQIP	2638	—	0.800 (0.750–0.850)	reported_CI
Moya A 2023	RAI	RAI	ACS-NSQIP	2864	—	0.741 (0.678–0.804)	reported_CI
Prvulovic S 2025	RAI	RAI	ACS-NSQIP	42230	—	0.820 (0.790–0.850)	reported_CI
Prvulovic ST 2025	RAI	RAI	NSQIP	360133	—	0.833 (0.819–0.847)	reported_CI
Rothenberg KA 2020	RAI	RAI	ACS-NSQIP 2007-2013	139569	—	0.720	reported_SE
Sabet A 2026	RAI	RAI	ACS-NSQIP	11965	19	0.595	estimated_SE
Sbaih O 2025	RAI	RAI	NSQIP	1637	—	0.790 (0.750–0.830)	reported_CI
Soni C 2025	RAI	RAI	ACS-NSQIP	206352	4745	0.840 (0.840–0.850)	reported_CI
Author not specified (Kaiser Permanente electronic frailty	mFI	mFI-5	KPNC (Kaiser Permanente Northern Ca	37186	—	0.640 (0.620–0.660)	reported_CI

First author, year	Tool family	Tool subtype	Data source	Sample size	30-day deaths	AUC (95% CI)	Variance source
metrics study) 2022							
Bowers C 2023	mFI	mFI-5	ACS-NSQIP	292225	—	0.677 (0.675–0.679)	reported_CI
Bowers C 2023	mFI	mFI-5	ACS-NSQIP	61441	—	0.684 (0.680–0.688)	reported_CI
Conlon M 2022	mFI	mFI-5	ACS-NSQIP	6571	—	0.622 (0.610–0.634)	reported_CI
Ehlert B 2016	mFI	mFI-11	ACS-NSQIP 2006-2012	72106	—	0.660 (0.630–0.700)	reported_CI
Ehlert B 2016	mFI	mFI-11	ACS-NSQIP 2006-2012	72106	—	0.630 (0.590–0.670)	reported_CI
Ehlert B 2016	mFI	mFI-11	ACS-NSQIP 2006-2012	72106	—	0.640 (0.600–0.690)	reported_CI
Gupta N 2025	mFI	mFI-5	NSQIP	169065	—	0.610 (0.608–0.612)	reported_CI
Gupta N 2025	mFI	mFI-5	ACS-NSQIP	114359	—	0.610 (0.600–0.620)	reported_CI
Hao Y 2025	mFI	mFI-5	single-center	699	21	0.790	estimated_SE
Hermann CE 2022	mFI	mFI-5	ACS-NSQIP 2015-2017	80293	—	0.819 (0.704–0.933)	reported_CI
Hermann CE 2022	mFI	mFI-5	ACS-NSQIP 2015-2017	80293	—	0.801 (0.750–0.851)	reported_CI
Hinojosa-Gonzalez D 2025	mFI	mFI-5	ACS-NSQIP (2019-2022)	7324	50	0.600	estimated_SE
Mau E 2025	mFI	mFI-5	ACS-NSQIP	53959	—	0.720 (0.710–0.730)	reported_CI
Modi M 2026	mFI	mFI-5	ACS-NSQIP	2638	—	0.610 (0.550–0.680)	reported_CI
Moya A 2023	mFI	mFI-5	ACS-NSQIP	2864	—	0.574 (0.489–0.659)	reported_CI
Musharbash FN 2023	mFI	mFI-5	ACS-NSQIP	1195	104	0.580	estimated_SE
Olgun Keleş G 2026	mFI	mFI-5	single-center	177	—	0.552 (0.431–0.673)	reported_CI
Prvulovic ST 2025	mFI	mFI-5	NSQIP	360133	—	0.658 (0.641–0.676)	reported_CI
Sabet A 2026	mFI	mFI-5	ACS-NSQIP	11965	19	0.601	estimated_SE
Sbaih O 2025	mFI	mFI-5	NSQIP	1637	—	0.570 (0.530–0.610)	reported_CI
Warrier A 2026	mFI	mFI-5	ACS-NSQIP	2862	—	0.722 (0.519–0.924)	reported_CI

Section B. Primary pooled AUC analysis

Table S4.2. Primary pooled AUC estimates for mFI and RAI.

Tool family	Rows (k)	Unique studies	Pooled AUC	95% CI	τ^2 (logit)	I^2	95% prediction interval (AUC)
mFI	22	19	0.646	0.616–0.675	0.0527	99%	0.527–0.750
RAI	22	19	0.791	0.761–0.819	0.1453	100%	0.627–0.895

Method: REML estimator for τ^2 with Hartung–Knapp confidence-interval adjustment on t-distribution with $k - 1$ degrees of freedom; back-transformed from the logit-AUC scale.

Section C. Sensitivity analysis excluding estimated standard errors

Table S4.3. Pooled AUC after excluding Hanley–McNeil estimated SE entries.

Of the 5 entries with Hanley–McNeil-imputed SE in the primary analysis, 4 were on the mFI side and 1 was on the RAI side. This sensitivity analysis re-pools each tool family after excluding all imputed-SE entries.

Tool family	Rows (k)	Unique studies	Pooled AUC	95% CI	τ^2 (logit)	I^2	95% prediction interval (AUC)
mFI	18	15	0.650	0.617–0.681	0.0527	99%	0.528–0.755
RAI	21	18	0.796	0.768–0.822	0.1293	100%	0.644–0.894

Section D. Overlap-adjusted sensitivity analysis

Table S4.4. Pooled AUC in the overlap-adjusted dataset.

The overlap-adjusted dataset retains one representative study per overlap group identified in Supplementary Appendix 3 (4 overlap groups; 7 studies excluded from the primary pool; 13 study-tool rows removed; 31 rows from 19 unique studies retained).

Tool family	Rows (k)	Unique studies	Pooled AUC	95% CI	τ^2 (logit)	I^2	95% prediction interval (AUC)
mFI	15	14	0.640	0.595–0.684	0.0697	84%	0.493–0.765
RAI	16	13	0.784	0.742–0.821	0.1749	100%	0.589–0.902

Section E. Tool subtype sensitivity analysis

Table S4.5. Tool subtype analyses.

Pooled estimates are reported only when at least 5 entries were available for a subtype; otherwise descriptive AUC values are summarised. mFI-5 and the original RAI subtypes had sufficient entries for pooled analysis. mFI-11, RAI-Rev, and RAI-A had too few entries for stable quantitative pooling and are reported descriptively.

Tool subtype	Rows	Unique studies	Pooled / median AUC	95% CI	τ^2 (logit)	I^2	Notes
mFI-5	19	18	0.648	0.611–0.683	0.0697	99%	Pooled (REML + HK)
mFI-11	3	1	0.640	—	—	—	Descriptive only (range 0.630–0.660)
RAI	16	16	0.778	0.740–0.812	0.1375	100%	Pooled (REML + HK)
RAI-rev	4	3	0.841	—	—	—	Descriptive only (range 0.720–0.870)
RAI-A	2	2	0.805	—	—	—	Descriptive only (range 0.768–0.842)

Section F. Supplementary CFS evidence

Table S4.6. AUC-ready CFS evidence.

CFS contributed only 5 AUC-ready entries; this is below the threshold considered adequate for stable pooled discrimination and CFS does not appear in any within-cohort head-to-head comparison against mFI or RAI in the included literature. CFS is therefore reported as supplementary narrative evidence and does not enter the primary pooled analysis.
Some supplementary CFS entries reported AUC point estimates without confidence intervals and were therefore summarized descriptively only.

First author, year	Journal	Tool subtype	AUC (95% CI)
Aakre E 2024	Anesthesia and Analgesia	CFS	0.690 (0.570–0.820)
Ikram MA 2022	The Bone & Joint Journal	CFS	0.631 (0.572–0.690)
Narula S 2020	Bone & Joint Open	CFS	0.699
Palaniappan M 2022	Colorectal Disease	CFS	0.710 (0.650–0.770)
Wu J 2021	International Urology and Nephrology	CFS	0.910 (0.840–0.970)
Summary across CFS entries	—	n = 5	median 0.699; range 0.631–0.910

Section G. Brief interpretation

In the primary AUC-ready dataset, the pooled discrimination of RAI for 30-day all-cause mortality after noncardiac surgery was 0.791 (95% CI 0.761–0.819), compared with 0.646 (95% CI 0.616–0.675) for mFI (between-tool absolute difference on the AUC scale: +0.145). Between-study heterogeneity was substantial for both pools (I^2 = 99% for mFI and 100% for RAI), consistent with the diversity of surgical specialties, tool subtypes, and cohort sizes represented in the included studies.

Excluding the 5 entries whose standard errors were imputed by the Hanley–McNeil method changed the pooled estimates by ≤ 0.005 AUC units in absolute terms and did not alter the qualitative ordering of RAI versus mFI; the pattern of higher pooled discrimination for RAI than for mFI was preserved.

In the overlap-adjusted dataset (one representative study retained per ACS-NSQIP overlap group), the pooled estimates were 0.640 (95% CI 0.595–0.684) for mFI and 0.784 (95% CI 0.742–0.821) for RAI (between-tool difference +0.143). The direction and approximate magnitude of the between-tool difference were preserved after overlap adjustment.

Tool-subtype analyses showed that mFI-5 and the original RAI subtypes contributed the bulk of evidence in their respective tool families. mFI-11, RAI-Rev, and RAI-A each had too few entries for stable quantitative pooling and are reported descriptively only. Differences in pooled discrimination between mFI-5 and the original RAI mirrored the parent tool-family comparison.

CFS contributed only 5 AUC-ready entries, with no within-cohort head-to-head data against mFI or RAI; pooled discrimination for CFS was therefore not estimated and CFS is reported as supplementary narrative evidence.

Exploratory small-study assessment was considered but interpreted cautiously because of the modest number of contributing studies and substantial between-study heterogeneity. Subgroup analyses by older-adult cohorts and elective versus emergency surgery, and a formal certainty-of-evidence appraisal adapted for prognostic studies, are summarised narratively because the available data were insufficient for stable quantitative pooling within those subsets.

End of Supplementary Appendix 4.
